# The Natural Product Corramycin Acts as a DNA Gyrase Poison and Overcomes Fluoroquinolone Resistance in *Mycobacterium tuberculosis*

**DOI:** 10.1002/advs.76832

**Published:** 2026-08-10

**Authors:** Franziska Fries, Jennifer Herrmann, Michael Dal Molin, Yaëlle Wormser, Timo Risch, Yunsheng Gao, Gareth Prosser, Francesca Gubellini, Ariel Mechaly, Joshua B. Wallach, Anthony Castro, Arka Banerjee, Sari Rasheed, Alexander Horn, Mathias Müsken, Michael Kurz, Lindsay Sonnenkalb, Viola Dreyer, Stefan Niemann, Sönke Andres, Dirk Schnappinger, Uli Kazmaier, Norbert Reiling, Rolf Müller, Jan Rybniker, Stéphanie Petrella

**Affiliations:** ^1^ Helmholtz Institute for Pharmaceutical Research Saarland (HIPS) Helmholtz Centre for Infection Research (HZI) and Department of Pharmacy Saarland University Saarbrücken Germany; ^2^ German Center for Infection Research (DZIF) Partner Site Hannover‐Braunschweig Braunschweig Germany; ^3^ Department I of Internal Medicine Faculty of Medicine and University Hospital Cologne University of Cologne Cologne Germany; ^4^ Center for Molecular Medicine Cologne (CMMC) University of Cologne Cologne Germany; ^5^ German Center for Infection Research (DZIF) Partner Site Bonn‐Cologne Cologne Germany; ^6^ Institut Pasteur Université Paris Cité CNRS UMR 3528 Bacterial Cell Cycle Mechanisms Unit Paris France; ^7^ Microbial Interface Biology Research Center Borstel Leibniz Lung Center Borstel Germany; ^8^ German Center for Infection Research (DZIF) Partner Site Hamburg‐Lübeck‐Borstel‐Riems Borstel Germany; ^9^ Institut Pasteur Université Paris Cité CNRS UMR 3528 Structural Microbiology Unit Paris France; ^10^ Institut Pasteur Plate‐Forme de Cristallographie – C2RT CNRS UMR 3528 Université Paris Cité Paris France; ^11^ Department of Microbiology and Immunology Weill Cornell Medical College New York New York USA; ^12^ Organic Chemistry I Saarland University Saarbrücken Germany; ^13^ Helmholtz Centre for Infection Research (HZI) Central Facility for Microscopy Braunschweig Germany; ^14^ Sanofi‐Aventis Deutschland R&D LGCR/Chemistry Industriepark Hoechst Frankfurt am Main Germany; ^15^ Molecular and Experimental Mycobacteriology Research Center Borstel, Leibniz Lung Center Borstel Germany; ^16^ National and WHO Supranational Reference Laboratory for Mycobacteria Research Center Borstel Leibniz Lung Center Borstel Germany

**Keywords:** cryo‐EM, DNA synthesis inhibitor, gyrase poison, mechanism of action, *Mycobacterium tuberculosis*, natural products

## Abstract

Despite significant advancements in drug development and discovery, tuberculosis remains one of the world's deadliest infectious diseases. The rise and global spread of multidrug‐resistant strains underscore a critical need for new antibiotics with distinct mechanisms of action. Screening of a myxobacterial extract library uncovered the previously unrecognized antitubercular activity of corramycin, a natural product with potent bactericidal activity against both drug‐sensitive and ‐resistant *Mycobacterium tuberculosis*. Corramycin enters the bacterial cell by exploiting multiple transport systems, and induces DNA double‐strand breaks through a novel form of DNA gyrase poisoning. The cryo‐electron microscopy structure of *M. tuberculosis* gyrase in complex with corramycin reveals that the natural product targets a site overlapping with the binding site of synthetic fluoroquinolones, thereby locking the gyrase in an inactive conformation and preventing re‐ligation of DNA. Importantly, its unique mode of binding allows corramycin to overcome fluoroquinolone resistance, highlighting its promise as a novel antibiotic scaffold to address the antimicrobial resistance crisis.

## Graphical abstract

Corramycin, a myxobacterial natural product antibiotic, exhibits potent bactericidal activity against multidrug‐resistant *Mycobacterium tuberculosis*. The compound hijacks bacterial transporters such as BacA and OppABCD to enter the cell and inhibits DNA synthesis through a previously unrecognized mode of DNA gyrase poisoning, locking the enzyme in an inactive conformation and thereby overcoming fluoroquinolone resistance.

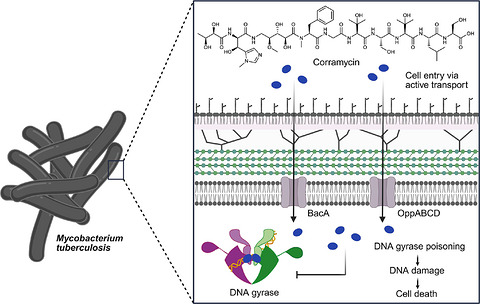

## 1 Introduction

Tuberculosis (TB) ranks among the world's deadliest infections and continues to pose a critical public health challenge. According to the World Health Organization (WHO), more than 10 million people fall ill with TB each year and in 2024, an estimated 1.23 million deaths were attributed to the disease, making TB once again the leading cause of death from a single infectious agent and one of the top 10 causes of death worldwide [1]. The currently recommended treatment regimen for drug‐sensitive (DS‐)TB, which involves a six‐month course of up to four antibiotics, is often complicated by adverse effects and poor adherence [2]. The situation is further exacerbated by the rise of multidrug‐resistant (MDR‐)TB, caused by *Mycobacterium tuberculosis* (*Mtb*) strains resistant to isoniazid and rifampicin, the two most important first‐line drugs. Of even greater concern is the emergence of extensively drug‐resistant (XDR‐)TB, characterized by additional resistance to fluoroquinolones (FQs) – key components in the treatment of MDR‐TB – as well as to either bedaquiline or linezolid [1, 3]. Reflecting the urgency of the crisis, the WHO has, for the first time, designated rifampicin‐resistant *Mtb* as a critical priority pathogen in its updated bacterial priority pathogens list [4]. These developments highlight the pressing need for novel antibiotics to address the growing threat of drug‐resistant (DR‐)TB.

Natural products from microbial, marine or plant origin, serve as a prolific resource for the discovery of new antibiotics [5, 6]. In recent years, there have been multiple reports of antitubercular natural products, featuring novel chemical scaffolds and innovative mechanisms of action such as griselimycins, which validated the DNA polymerase III sliding clamp (DnaN) as an intriguing new target [7], or amycobactin, the first compound to inhibit the mycobacterial Sec protein machinery [8].

Corramycin represents another promising natural product antibiotic, comprising a novel chemical scaffold. Initial characterization of its anti‐infective properties revealed a narrow antibacterial spectrum, targeting Gram‐negative bacteria from the *Enterobacteriaceae* family, while sparing Gram‐positive pathogens. Importantly, corramycin showed outstanding efficacy in an in vivo proof‐of‐concept study in rodents, highlighting its potential for drug development [9, 10]. Renard et al. previously described a multiparametric lead optimization program for the application of corramycin in urinary tract infections (UTI). Employing a Trojan horse strategy, they identified a preclinical candidate with improved mouse pharmacokinetics (PK) and a broader antibacterial spectrum along with significantly improved in vitro and in vivo potencies [11]. Of note, these optimizations toward a candidate for UTI treatment were performed in the absence of knowledge of the molecular target and the mode of action (MOA) of corramycins.

Here, we demonstrate that corramycin exhibits activity in the sub‐ to low‐µM range against the critical priority pathogen *Mtb*, including DR isolates, and we identify and validate DNA gyrase as its primary target in *Mtb*. Notably, the cryo‐electron microscopy (cryo‐EM) structure of *Mtb* DNA gyrase in complex with corramycin reveals the molecular basis of target engagement and uncovers a previously unrecognized mode of inhibition.

## 2 Results

### 2.1 Myxobacterial Extract Screening Identifies Corramycin as a Bactericidal *Mtb* Inhibitor

**FIGURE 1:**
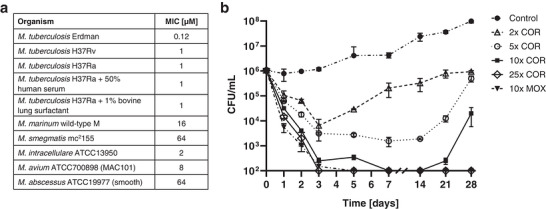
In vitro activity of corramycin. (a) Minimum inhibitory concentrations (MICs) against selected *Mycobacterium* species. Values represent a minimum of two independent repeats. (b) Time‐ and concentration‐dependent killing of *Mycobacterium tuberculosis* H37Ra by corramycin. Limit of detection is 10^2^ CFU/mL. Data represent mean ± SD of two technical replicates (*n* = 2). COR, corramycin; MOX, moxifloxacin.

To identify new antitubercular hits, we screened a proprietary collection of 2,552 crude extracts from myxobacteria, which were collected as part of natural compound libraries of the German Center for Infection Research (DZIF) [12]. Screening against *Mtb* Erdman in resazurin reduction microtiter assays (REMA) resulted in a primary confirmed hit rate of 4.0%, i.e., 102 extracts of the collection showed potent and reproducible *Mtb* inhibitory properties. Next, all hit extracts were analyzed for the presence of known compounds based on HPLC/high‐resolution mass spectrometry (HRMS) data. Dereplication using our in‐house database system Myxobase led to the exclusion of 46 crude extracts containing pan‐actives (e.g., saframycins [13]) and natural products with known anti‐*Mtb* activity (e.g., sorangicins [14]). From the 56 remaining hits, the crude extract of strain *Corallococcus coralloides* MCy10984 appeared particularly promising as it showed potent activity not only against *Mtb* Erdman but also against corresponding isoniazid‐ and rifampicin‐resistant strains, and no toxicity in HepG2 cells. This myxobacterial strain was recently described to produce the natural product corramycin, a peptidic antibiotic so far only known to exhibit a narrow spectrum of activity, mainly against *Escherichia coli* strains, with no detectable cytotoxicity across a panel of mammalian cell lines (Table S1) [9]. Intrigued by this unexpected finding, we validated that the activity of the MCy10984 crude extract stemmed from corramycin by testing fractionated crude extracts against *Mtb*, where we could clearly link anti‐*Mtb* activity with the presence of corramycin. Testing of the purified compound against various species from the *Mycobacterium* genus revealed that corramycin is most potent against *Mtb* with a minimum inhibitory concentration (MIC) in the sub‐ to low‐µM range (Figure 1a). This activity was retained upon addition of 50% human serum and 1% bovine lung surfactant. In addition, the compound showed good to moderate activity against *M. marinum* and members belonging to the *M. avium* complex (MAC). Much lower activities were observed against the fast‐growing *M. smegmatis*, and against *M. abscessus*, rendering corramycin highly selective for *Mtb*. Furthermore, we tested the natural product against *Mtb* strains from different lineages and with different drug resistance profiles, including MDR clinical isolates and strains resistant to bedaquiline. Corramycin showed similar MICs on all *Mtb* strains tested, demonstrating a lack of cross‐resistance with many drugs in clinical use (Table S2). Despite its potent in vitro activity against extracellular *Mtb*, corramycin displayed only moderate concentration‐dependent activity against intracellular bacilli. While intracellular growth was restricted by up to 75–80%, substantially higher concentrations (16x MIC) were required compared with rifampicin, which reduced intracellular bacterial levels by more than 90% at concentrations close to its MIC (Figure S1).

The killing kinetics of corramycin against *Mtb* revealed time‐ and concentration‐dependent bactericidal activity in vitro (Figure 1b and Figure S2). Concentrations of 2x and 5x MIC temporarily led to a 2 log_10_ reduction in colony‐forming units (CFUs), whereas concentrations of 10x and 25x MIC resulted in a more pronounced effect with a reduction of > 3 log_10_ units of the original count, demonstrating bactericidal activity [15]. While treatment with 10x MIC resulted in a re‐growth of mycobacteria after 14 days, 25x MIC completely eradicated *Mtb* with activity comparable to 10x MIC of moxifloxacin (Figure 1b), a second‐line antibiotic used for treatment of MDR‐TB [16]. Notably, a similar concentration‐dependent killing profile was observed in the *Mtb* Erdman strain (Figure S2), supporting the reproducibility of this bactericidal phenotype across distinct *Mtb* strains.

### 2.2 Corramycin Inhibits DNA Replication

**FIGURE 2:**
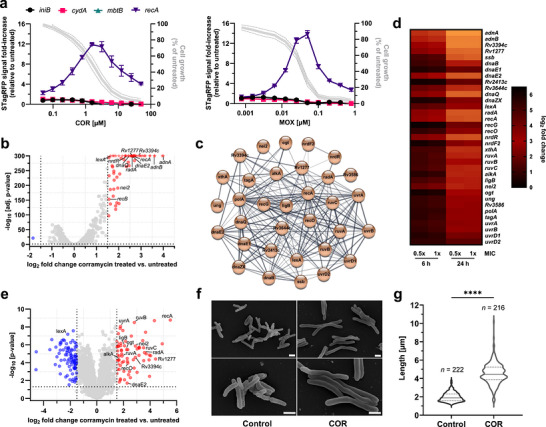
Corramycin induces the DNA damage response in *Mycobacterium tuberculosis*. (a) Fluorescent reporter assay showed that corramycin selectively promotes expression of the *recA* promotor in *Mtb*. Moxifloxacin was used as positive control for the *recA* reporter strain. Positive controls for the other reporters can be found in Figure S3. Data represent mean ± SD of two biological replicates (*n* = 2). (b) Transcriptome analysis of *Mtb* exposed to sub‐MIC levels of corramycin (0.5x MIC for 6 h) revealed up‐regulation of SOS response clusters and several DNA repair‐associated genes under the control of the PafBC regulon. Volcano plots show RNAs with significant changes in expression (log_2_ fold change ≥ 1.5, *p*‐value < 0.05). Significance was determined by the Wald test (*n* = 3). Each point represents the average value of one transcript. Asterisks represent imputed values for which the p‐value was recorded as 0. (c) Network cluster of genes involved in the DNA damage response, identified in the full STRING network of up‐regulated genes (1x MIC, 24 h). (d) Heat map of differentially expressed genes belonging to the local STRING network shown in (c). (e) Proteomics of *Mtb* exposed to sub‐MIC levels of corramycin (0.125x MIC until stationary phase). Volcano plots show proteins with significant changes in expression (log_2_ fold change ≥ 1.5, *p*‐value < 0.05). Significance was determined by two‐tailed student's T‐test (*n* = 4). Each point represents the average value of one protein. (f) Scanning electron microscopy (SEM) of *Mtb* exposed to 5x MIC of corramycin (T = 3 d). Scale bars are 1 µm. (g) Violin plot showing cell elongation in the presence of 5x MIC of corramycin. Significance was determined by two‐tailed Mann‐Whitney U test. *p* < 0.0001: ****. COR, corramycin; MOX, moxifloxacin.

To gain insights into the MOA, we monitored the activity of corramycin in a fluorescent reporter assay allowing the detection of iron deprivation (*mbtB*) [17] as well as disruption of cell wall biosynthesis (*iniB*) [18], DNA metabolism (*recA*) [18] and mycobacterial respiration (*cydA*) [19]. Like the FQ moxifloxacin, corramycin triggered dose‐dependent expression of the *recA* promotor, while other reporters were not induced (Figure 2a and Figure S3). To validate this finding, we conducted transcriptome and proteome analyses of *Mtb* exposed to corramycin. The natural product induced a characteristic transcriptomic and proteomic signature that featured up‐regulation of genes and proteins involved in DNA damage response pathways (Figure 2b,e). This comprised activation of the LexA/RecA‐dependent SOS response, corroborated by an up‐regulation of the recombinase RecA and the error‐prone polymerase DnaE2, and down‐regulation of the LexA repressor (Figure 2e). Furthermore, various components of the PafBC‐mediated DNA damage response pathway were up‐regulated, including *adnAB*, *recB*, *dnaE1*, *dnaQ*, *ruvA*, *nei2* and *polA*, which play diverse roles in base and nucleotide excision repair (BER and NER), homologous recombination, and DNA replication (Figure 2c,d) [20]. This physiological response closely resembles that observed in bacteria treated with genotoxins known to cause DNA double‐strand breaks (DSBs), such as H_2_O_2_, mitomycin C, or FQs [21, 22, 23]. However, similar responses can also arise from replication fork perturbation as seen for the DnaE1 inhibitor nargenicin [21, 24].

Through scanning electron microscopy (SEM) imaging, we observed that *Mtb* cells were elongated approximately 2‐ to 3‐fold (*p* < 0.0001) in the presence of corramycin (Figure 2f,g). This morphological change is frequently induced by DNA synthesis inhibitors such as gatifloxacin, novobiocin, or mitomycin C [25, 26]. Thus, corramycin likely acts by inhibiting DNA replication. We did not detect any direct interaction with DNA through intercalation or minor groove binding (Figure S4), indicating that the natural product most likely targets a protein involved in DNA metabolism rather than directly interacting with the DNA itself.

Transcriptional and proteome analyses in *E. coli* along with SEM imaging revealed a similar pattern of DNA stress (Figure S5), suggesting that the MOA of corramycin is likely consistent between *Mtb* and *E. coli*.

### 2.3 Corramycin Targets the DNA Gyrase and Exploits Different Transport Systems to Enter the Cell

**FIGURE 3:**
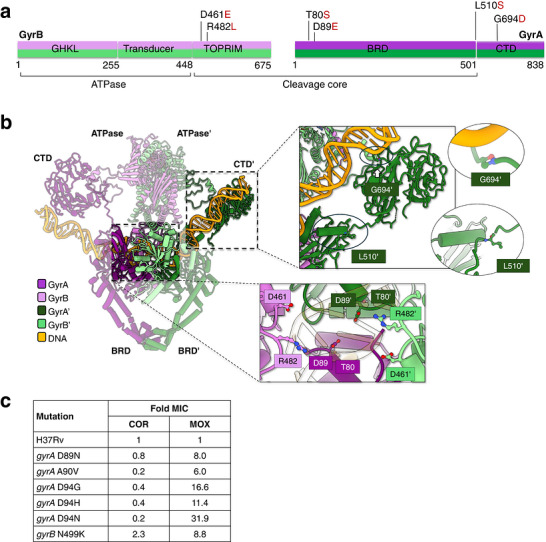
Resistance studies identify DNA gyrase as the target of corramycin in *Mycobacterium tuberculosis* and reveal absence of cross‐resistance in moxifloxacin‐resistant strains. (a) Domain architecture of DNA gyrase with indicated positions for mutations conferring corramycin resistance. (b) Mutation sites mapped onto the structure of the *Mtb* DNA gyrase suggest a possible binding site of corramycin inside the cleavage core. The structure is a model of the *Mtb* H37Rv gyrase using all crystallographic structures already published (PDB: 4G3N [34], 6GAV [35], and 3ZKD [36]), and was prepared in ChimeraX. (c) Relative shifts of corramycin activity against FQ‐resistant *Mtb* strains. Fold MICs are based on the wild‐type strain *Mtb* H37Rv. BRD, breakage‐reunion domain; CTD, C‐terminal domain.

Previous efforts to identify the molecular target of corramycin through spontaneous resistance in *E. coli* failed as they only led to emergence of importer mutations [9]. For *Mtb*, we therefore performed extensive experiments using different methods based on both liquid and solid media, which gave rise to resistant mutants at a low frequency of 6.56 × 10^−9^. Whole genome sequencing (WGS) of these mutants revealed resistance conferring variants in *gyrA* (D89E) and *gyrB* (R482L), encoding the two DNA gyrase subunits A and B, respectively. To enforce further mutations, we exposed *Mtb* with the *gyrA* D89E background to even higher corramycin concentrations. This led to the identification of additional mutations in the *gyrB* gene (R482L and D461E) (Figure 3a and Table S3). Of note, presence of both *gyrA* and *gyrB* mutations caused high‐level corramycin resistance with up to 300‐fold shifts in MIC (Table S4). To rule out that other spontaneous mutations in non‐gyrase genes induced corramycin resistance, we introduced the *gyrB* D461E and R482L, as well as the *gyrA* D89E mutations into wild‐type *Mtb* by recombineering [27]. The recombineered strains showed resistance patterns consistent with those expected from the introduced mutations, with MIC shifts comparable to those of spontaneous mutants carrying the same variants (Table S4).

Using a serial passaging approach (Figure S6), we identified even more gyrase mutations conferring corramycin resistance. Four mutants were found to contain mutations in *gyrA* (T80S, D89E, L510S and G694D), and one in *gyrB* (R482L) (Figure 3a and Table S5). Importantly, gyrase mutations leading to corramycin resistance did not result in cross‐resistance with the FQs moxifloxacin and ciprofloxacin (Table S6). Likewise, *gyrA* and *gyrB* mutations, accounting for over 80% of clinical resistance to FQs [28], had no or very little impact on corramycin MICs in a laboratory *Mtb* strain (Figure 3c), and corramycin similarly retained activity against FQ‐resistant *Mtb* clinical isolates (Table S2), suggesting a mode of DNA gyrase engagement distinct from that of FQs. Consistent with these findings, corramycin has previously been shown to remain active against FQ‐resistant MDR *E. coli* clinical isolates [10], indicating that the absence of cross‐resistance may extend beyond *Mtb*.

Bacterial type II topoisomerases are ubiquitous enzymes that regulate DNA topology by passing a DNA duplex through a transient double‐strand break [29, 30]. While most bacterial species encode topoisomerase IV in addition to gyrase, the *Mycobacterium* genus lacks topo IV [31], requiring DNA gyrase to take over its critical biological functions [32]. The DNA gyrase is a heterotetrameric enzyme (A_2_B_2_) composed of two GyrA and GyrB subunits, respectively [33]. While the mutations GyrA G694D and L510S occur in the distinct C‐terminal domain (CTD) and in the linker region connecting the breakage‐reunion domain (BRD) to the CTD, the other four mutation sites are located in close proximity within the cleavage core, hinting toward a potential binding site of corramycin (Figure 3b).

We additionally detected mutations in the transport systems BacA and OppABCD (Table S5). BacA is a transmembrane protein transporter involved in vitamin B_12_ uptake and has been shown to function as a multi‐solute transporter for hydrophilic molecules [37, 38]. In line with this, BacA mutants displayed increased resistance to bleomycin, a known BacA substrate [38], confirming functional impairment of the transporter in resistant *Mtb*. Importantly, no resistance was observed to any first‐, second‐, or last line anti‐*Mtb* drugs (Table S6). Phylogenetic studies indicate that BacA homologs are distributed among other bacteria, sharing high conservation of function [39, 40]. Notably, corramycin was already shown to exploit the homolog SbmA to enter *E. coli* [9], and other molecules were reported to employ the same uptake mechanism, resulting in a high degree of selectivity for certain bacterial species [41, 42, 43, 44]. Mutations in *bacA* were consistently accompanied by mutations in different subunits of the oligopeptide permease OppABCD (Table S5). The latter belongs to the type I ABC transporter family and is known to serve as an oligopeptide importer with a wide range of substrates, including glutathione and bradykinin [45, 46]. Interestingly, WGS of mutants harvested at a lower corramycin selective pressure revealed that gyrase mutations appear to arise first (Figure S6 and Table S5). We hypothesize that this may result from transporter redundancy, requiring simultaneous mutation events in both transport systems for corramycin resistance to develop, whereas for *gyrA* and *gyrB* a single point mutation is sufficient (Tables S4–S6).

### 2.4 Corramycin is a Bacterial DNA Gyrase Poison

**FIGURE 4:**
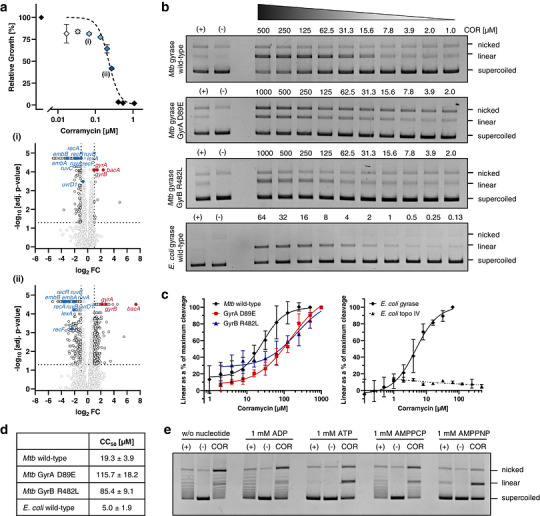
Corramycin acts as a DNA gyrase poison. (a) CRISPRi screen identified *bacA* and *gyrAB* as resistor genes. Relative growth of *Mycobacterium tuberculosis* upon corramycin exposure and volcano plots showing genes that influence corramycin efficacy in *Mtb* at indicated drug concentrations ((i) 0.125x MIC, (ii) 0.5x MIC). Significant hits (log_2_ fold change (FC) ≥ 1, *p*‐value < 0.05, *n* = 3) were colored in black. Genes of interest are colored in blue (sensitizers) and red (resistors). An overview of all screened drug concentrations is shown in Figure S7. (b) Cleavage assays for wild‐type (47 nM) and mutant *Mtb* (GyrA D89E: 80 nM, GyrB R482L: 213 nM) and *Escherichia coli* (107 nM) gyrases. The positions of nicked, linear and supercoiled plasmid are indicated. A representative agarose gel is shown. (c) Plots used for CC_50_ (concentration required to achieve half of maximum DNA cleavage) determination. Values are plotted as mean values ± SD (*n* = 3). Lines represent sigmoidal fits to the data. Agarose gel depicting the *E. coli* topoisomerase IV cleavage assay can be found in Figure S9. (d) CC_50_ values of corramycin determined for wild‐type *E. coli* and *Mtb* gyrase, and for mutant *Mtb* gyrase. (e) ATP dependency of corramycin‐mediated DNA cleavage. Agarose gel of an *Mtb* gyrase DNA cleavage reaction with 100 µM corramycin and 47 nM *Mtb* gyrase in the absence and presence of a nucleotide (ADP, ATP, AMPPCP and AMPPNP) is shown. (+) represents a positive control containing gyrase without inhibitor, while (‐) represents a negative control without gyrase and without inhibitor. COR, corramycin.

Previous work suggested DNA gyrase as potential target of corramycin, but the only moderate inhibition of *E. coli* gyrase supercoiling activity (IC_50_ 27 µM) [47] relative to its MIC (0.5‐8 µM) left the role of gyrase inhibition unresolved, and the target hypothesis was not pursued further, with no additional evidence establishing gyrase as the primary target in *E. coli*. However, the resistance mutations identified in *Mtb* along with the associated high MIC shifts strongly support DNA gyrase as the primary target of corramycin (Tables S4 and S6). To assess the mechanism of corramycin further, we applied a genome‐wide CRISPRi library to identify the gene products that determine potency of corramycin against *Mtb*. This identified *bacA*, *gyrA* and *gyrB* to be among the genes that upon silencing increase fitness of *Mtb* during growth in sub‐MIC concentrations of corramycin (Figure 4a and Figure S7). That silencing of *bacA* increased resistance of *Mtb* to corramycin further reinforces its role in facilitating cytoplasmic entry. Resistance conferred by silencing of *gyrA* and *gyrB* supports engagement of gyrase by corramycin and suggests poisoning of gyrase to be the primary MOA. Gyrase poisons such as FQs trap gyrase‐DNA cleavage complexes, preventing DNA re‐ligation and thereby generating lethal DSBs. A key consequence is that toxicity scales with the number of active gyrase molecules engaging DNA, as drug‐stabilized cleavage complexes form proportionally to target abundance. Accordingly, reduced expression of *gyrA* and *gyrB* limits formation of these complexes and mitigates DNA damage, whereas increased target levels sensitize cells to gyrase poisons [48]. This is consistent with corramycin acting as a gyrase poison and contrasts with classical catalytic inhibitors such as aminocoumarins, which block the ATPase activity of GyrB and for which target depletion typically increases sensitivity rather than conferring resistance. This interpretation is further supported by sensitization of *Mtb* to growth inhibition by corramycin in response to silencing of many gene products involved in DNA repair, such as *ruvABC*, *lexA*, *recF*, *recR*, and *uvrD1*. Moreover, silencing of *embA* and *embB* increased susceptibility of *Mtb* to corramycin, which is in accordance with a weak synergy observed between corramycin and ethambutol (Figure S8).

Supporting the MOA hypothesis derived from the CRISPRi profile, we found that corramycin stabilizes double‐strand broken DNA bound to gyrase as evidenced by the appearance of linear cleavage products (Figure 4b). By using a fixed amount of DNA gyrase and titrating corramycin in the presence of ATP, we determined the CC_50_ (concentration required to achieve half of maximum DNA cleavage) values to be 19.3 ± 3.9 and 5.0 ± 1.9 µM for *Mtb* and *E. coli* gyrases, respectively. We further tested corramycin on the purified gyrase variants carrying the mutations identified in both *Mtb* H37Ra and *Mtb* Erdman, and found that GyrA D89E as well as GyrB R482L result in reduced corramycin‐induced DNA cleavage (Figure 4c,d). Surprisingly, even at high concentrations, corramycin did not promote DNA cleavage by *E. coli* topo IV (Figure 4c and Figure S9). Thus, the compound appears to be a selective inhibitor of DNA gyrase. Furthermore, we found that corramycin activity was nucleotide‐dependent. Little to no double‐strand DNA cleavage was observed in the absence of a nucleotide or in the presence of ADP. In contrast, ATP promoted robust cleavage, and even stronger cleavage was detected with the non‐hydrolyzable ATP analog adenosine 5’‐(*β*,*γ*‐imido)triphosphate (AMPPNP). Interestingly, cleavage was reduced in the presence of a second non‐hydrolyzable analog, adenosine 5’‐(*β*,*γ*‐methylene)triphosphate (AMPPCP) (Figure 4e). While ATP and AMPPNP both stabilize a dimeric, closed conformation of the ATPase domains, AMPPCP predominantly maintains a monomeric state [36]. These data suggest that corramycin activity is favored by ATPase domain dimerization.

Given the high conservation of the DNA gyrase across different bacterial species, we further explored the ability of corramycin to stimulate DNA cleavage by the *Staphylococcus aureus* gyrase. *S. aureus* as a Gram‐positive pathogen is not susceptible to corramycin (Table S1), yet we found the agent to stabilize DSBs to an extent similar to that of *E. coli* gyrase (CC_50_ 4.6 ± 2.1 µM) (Figure S10). This finding demonstrates that the missing in vitro activity against Gram‐positive bacteria is most likely due to the absence of a suitable importer rather than the lack of the target in these pathogens.

### 2.5 Corramycin Locks *Mtb* Gyrase in Its Post‐Cleavage Conformation

**FIGURE 5:**
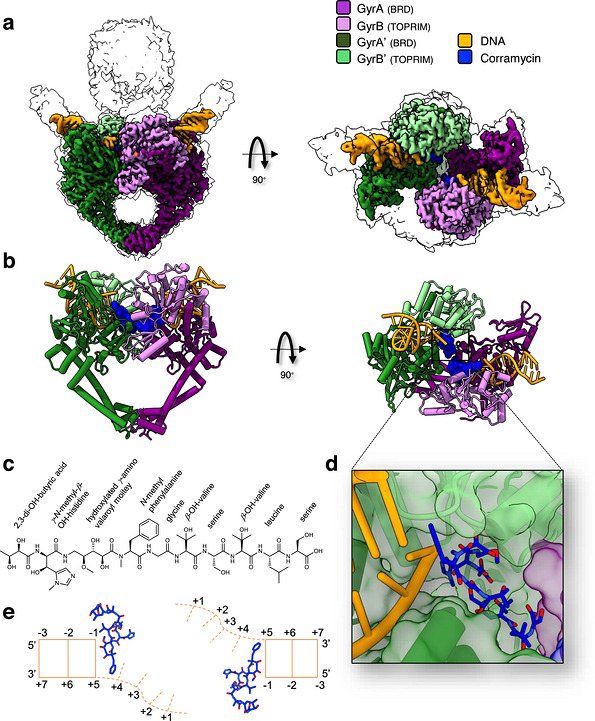
Cryo‐EM visualization of corramycin binding pockets within the *Mycobacterium tuberculosis* DNA gyrase. (a) The gyrase/DNA/COR cryo‐EM map at two different contour level maps. The low‐resolution contour (white) shows the positioning of the GyrB ATPase domains and the absence of density for the GyrA CTDs. The high‐resolution contour is color‐coded with GyrA and GyrA’ in dark magenta and forest green, GyrB and GyrB’ in medium orchid and light green, corramycin in blue and DNA in orange. (b) A model overview of the gyrase/DNA/COR complex. GyrA (I15‐I501) and GyrB (K441–A667) subunits are depicted as cartoon representation (cylinders/stubs) with the same color scheme as panel (a). A 12‐bp double‐stranded, cleaved DNA segment is shown in orange. Two corramycin molecules are shown as molecular surface representation. (c) Chemical structure of corramycin. (d) A detailed view of the corramycin binding pocket composed of GyrA, GyrA’, GyrB and DNA. The gyrase is displayed as molecular surface, DNA as cartoon representation, and corramycin as stick representation. (e) Schematic representation of how corramycin (blue stick representation) interacts with the ‐1 base positions on both strands at sites of DNA cleavage. BRD, breakage‐reunion domain; CTD, C‐terminal domain.

The determination of the structure of the *Mtb* gyrase in complex with corramycin by cryo‐EM allowed the characterization of its binding mode. For this part of the study, we employed a fusion construct of the gyrase, termed *Mtb* GyrBA. Biochemical characterization demonstrated that the fused enzyme retained catalytic activity comparable to that of the individual subunits [49] and remained fully susceptible to corramycin inhibition, with a CC_50_ value closely matching that observed for the non‐fused form (Figure S11). The collected data feature the *Mtb* GyrBA fusion in complex with 150 bp double‐stranded DNA (dsDNA), the nucleotide analog AMPPNP, and corramycin under saturating conditions. A cryo‐EM map of the gyrase cleavage core, encompassing residues K441‐A667 of GyrB and I15‐I501 of GyrA, was obtained at an overall resolution of 2.80 Å following several rounds of 3D classification (Figure 5 and Figure S12, and Table S7). This map allowed the unequivocal identification of all secondary structure elements and amino acid residue side chains as well as the reconstruction of two segments of dsDNA bound inside the groove of the cleavage core (Figure 5 and Figure S13). In addition, the low‐resolution contour for this map enabled the visualization of the entire enzyme‐DNA complex and revealed a dimeric conformation for the ATPase domains (Figure 5a).

We clearly identified two corramycin molecules as distinct density regions (Figure 5 and Figure S13f), localized near the proposed binding site within the gyrase cleavage core, as suggested by mutation analysis (Figure 3a,b). Similar to FQs, corramycin binds to the enzyme in a symmetric manner (Figure 5a,b,e). Comparison of the *Mtb* gyrase cleavage core structures bound to corramycin, FQs (represented by moxifloxacin, PDB: 5BS8), novel bacterial topoisomerase II inhibitors (NBTIs, represented by BDM71403, PDB: 8S7O) and evybactin (PDB: 7UGW) revealed a distinct conformational state induced by corramycin. The *Mtb* gyrase cleavage core in the FQ‐, NBTI‐ and evybactin‐bound complexes consistently shows a closed conformation. In contrast, in the presence of corramycin, the cleavage core adopts an open conformation, with dsDNA accommodated inside (Figure S14). This open state is accompanied by an increased spacing between the two opposing helices α3 and α3′ (residues 74–84) at the GyrA/GyrA′ dimer interface, compared to the BDM71403‐bound complex. Specifically, the distance between the aforementioned helices increases by 5 Å, indicating a significant opening of the enzyme. Additionally, a 30° shift is observed in the GyrB TOPRIM domain.

Another significant difference to FQ‐ and NBTI‐bound complexes is the disruption of base pairing for the dsDNA (Figure S13a–c). The dsDNA is cleaved on each strand, a state typically observed in gyrase‐FQ complexes [50, 51]. However, the key difference is that the catalytic tyrosine (Y_cat_129) is not covalently bound to the scissile phosphate at the +1 position on the 5’‐end of the dsDNA. Structural alignment of the *Mtb* gyrase cleavage cores bound to corramycin and moxifloxacin reveals that Y_cat_ residues of GyrA in each complex are not superposable (Figure S13d,e,g). Additionally, the four nucleotides located near the corramycin molecules (+1 to +4) become highly disordered upon opening of the cleavage core, leading to disruption of base‐pair interactions (Figure 5e and Figure S13).

### 2.6 Corramycin Binds to an Overlapping Pocket Shared by Fluoroquinolones

**FIGURE 6:**
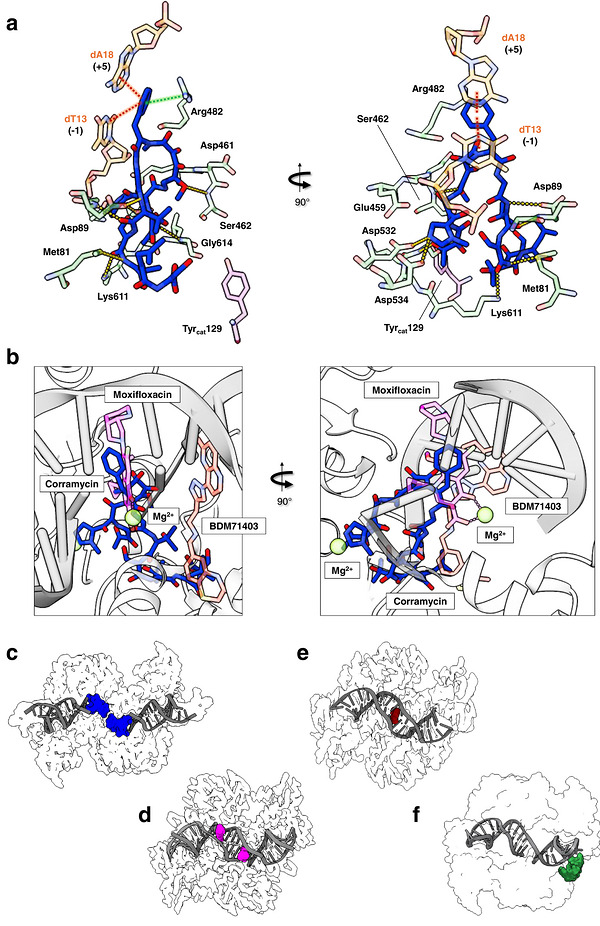
Binding pocket of corramycin. (a) The molecular interactions of corramycin rationalize the observed mutation sites. Interactions between GyrA‐GyrB and corramycin are illustrated using stick representations. Residues from GyrA are colored in forest green, from GyrB in light green, from the GyrA’ subunit in magenta, and DNA is shown in orange. Hydrogen bonds are colored in yellow, π–π interactions in orange and cation‐π interactions in green. (b) Comparative view of the binding sites of corramycin (current study), moxifloxacin (MOX) (PDB: 5BS8), and BDM71403 (PDB: 8S7O) on the GyrA‐GyrB DNA‐binding surface. Corramycin is displayed in blue, MOX in magenta and BDM71403 in red stick representations. Part of corramycin shares its binding pocket with MOX; however, unlike MOX, it does not intercalate between two base pairs, and it is not stabilized by an Mg^2+^ ion. (c–f) *Mycobacterium tuberculosis* gyrase in complex with corramycin (blue), MOX (magenta), BDM71403 (red) and evybactin (green, PDB: 7UGW). The map is shown in white and the DNA in dark gray.

The binding pocket of corramycin partially overlaps with that of FQs, yet the two compounds engage different residues and adopt fundamentally distinct binding modes (Figure 6b–d). This is consistent with the observed lack of cross‐resistance to this class of antibiotics (Figure 3c, Tables S2 and S6). Moreover, the corramycin binding pocket is entirely distinct from that of NBTIs, differing both in terms of location and induced state of the cleavage core, indicating that corramycin and NBTIs bind in a mutually exclusive manner (Figure 6b,c,e). Likewise, the recently identified natural product evybactin engages a completely separate pocket (Figure 6f).

Notably, corramycin binds to the *Mtb* gyrase in a *β*‐hairpin‐like conformation, similar to that observed in the high‐resolution crystal structure of the drug in complex with the kinase ComG (PDB: 8AGY), the self‐resistance factor of the producer *C. coralloides* (Figure 5d and Figure S15) [10]. Intrigued by this finding, we determined corramycin's 3D solution structure using nuclear magnetic resonance (NMR)‐based molecular dynamics (MD) simulations. The resulting conformation closely matched the folds observed in both ComG‐ and gyrase‐bound complexes, suggesting that the *β*‐hairpin represents the most stable and predominant conformation of the molecule. A common feature across all three structures is that the *N*‐methyl‐phenylalanine residue constitutes the turn of the hairpin, dividing the molecule into a western and eastern part, which are stabilized by intramolecular hydrogen bonds (Figure S15).

Corramycin is further stabilized within its binding pocket by an extensive network of hydrogen bonds involving the main chains and side chains of several amino acid residues from both the GyrA and GyrB subunits as well as the dsDNA (Figure 6a and Table S8). In the western half of the molecule, the *α*‐hydroxyl group of the hydroxylated *γ*‐amino valeroyl moiety forms a hydrogen bond with the backbone nitrogen of GyrB D461 (Figure 6a and Table S8), which emerged as one of the mutation sites linked to corramycin resistance (Figure 3a). Moreover, the *γ*‐*N*‐methyl‐*β*‐OH‐histidine seems to be central to the binding process. Its *β*‐hydroxyl group forms an intramolecular hydrogen bond with the side chain hydroxyl group of the corramycin serine flanked by the two *β*‐OH‐valines (Figure S15), a feature we consider essential for anchoring the imidazole ring. Furthermore, the protonated imidazole nitrogen establishes hydrogen bonds with the carboxylate oxygens of GyrB E459, D532 and D534 (Figure 6a and Table S8). Notably, the biosynthetic uniqueness of the *γ*‐*N*‐methyl‐*β*‐OH‐histidine moiety is mirrored by its importance in corramycin function as it occupies the position typically held by the active site catalytic Mg^2+^ ions within the highly hydrophilic pocket formed by the residues E459, D532 and D534 (Figure 6a,b). This finding aligns with the lack of density for Mg^2+^ ions in the gyrase‐corramycin complex. The eastern part of corramycin is also largely stabilized by hydrogen bonds, involving GyrA and GyrB residues such as GyrA D89, another key residue associated with corramycin resistance (Figure 6a and Table S8). The most striking aspect of corramycin binding comprises the π‐π stacking between the phenyl group of the *N*‐methyl‐phenylalanine and the nucleotide base at the 3’‐OH of the cleaved DNA (+5 dA18) (Figures 5e and 6a). On the opposite side, the phenyl group engages in a cation‐π‐interaction with the guanidinium group of the GyrB subunit R482 side chain (Figure 6a). Together, these interactions contribute to the anchoring of the cleaved DNA to the GyrB TOPRIM domain, even in absence of Mg^2+^ ions and in absence of the covalent phosphotyrosine bond.

Consistent with the lack of metal ions in the gyrase‐corramycin complex, we found that corramycin‐induced DNA cleavage was not reversed by addition of ethylenediaminetetraacetic acid (EDTA), suggesting a remarkably stable cleavage complex and confirming an Mg^2+^‐independent mechanism of inhibition (Figure S16). In contrast, EDTA efficiently resealed FQ‐stabilized DNA cleavage within less than 1 min for ciprofloxacin and within 5 min for moxifloxacin, most likely through disruption of the water‐metal ion bridge essential to the FQ ternary complex. Concomitant with the disappearance of the linear DNA product, we observed a transient increase in nicked (single‐strand broken) DNA, indicating that resealing proceeds through a nicked intermediate (Figure S16). Together, these findings highlight an additional mechanistic distinction between corramycin and FQs, with corramycin forming an unusually stable, Mg^2+^‐independent cleavage complex.

## 3 Discussion

In this study, we reveal that the antibiotic corramycin shows potent inhibitory activity against clinically relevant DS and DR *Mtb* strains. Exploiting a combination of ‐omics approaches, microbiological and biochemical assays, we provide the first in‐depth characterization of corramycin's MOA, which had thus far remained elusive. Our comprehensive approach identified and validated DNA gyrase, a crucial enzyme involved in bacterial DNA replication, as the primary target of this natural product in *Mtb*. To gain high‐resolution structural insights into the corramycin‐gyrase interaction, we performed single particle cryo‐EM analysis, allowing us to decipher its MOA more than a decade after its discovery.

Corramycin exhibits a distinctive MOA on DNA gyrase, distinguishing it from other known inhibitors such as FQs, NBTIs or novel *Mtb* gyrase inhibitors like evybactin, which binds to a distinct allosteric site [44, 49, 50, 51]. Accordingly, corramycin does not exhibit cross‐resistance to FQs and NBTIs. Corramycin engages a transient binding pocket at the interface of GyrA, GyrB, and DNA, which forms during the conformational changes associated with the gyrase catalytic cycle. Similar to established gyrase poisons such as FQs, the compound binds symmetrically to the enzyme, interacting with both the protein components and dsDNA. However, unlike FQs, corramycin does not stabilize the dsDNA through base‐pair intercalation, and no covalent phosphotyrosine bond between GyrA and the cleaved DNA is observed. Instead, anchoring of the DNA is achieved by an asymmetric interaction: one side of the phenyl group of corramycin binds cleaved dsDNA, while the opposing side of the ring engages the protein part of the complex. Strikingly, the antibiotic locks the gyrase cleavage core in a previously unseen post‐cleavage open conformation (Figure S17). When the pocket formed by cleaved DNA and gyrase residues becomes accessible, two corramycin molecules bind, preventing the “sliding door” movement in both directions. Additionally, corramycin binding displaces the catalytic Mg^2+^ ion, essential for re‐ligating the dsDNA. Without this ion anchoring of the nucleophilic 3’OH group of the cleaved DNA to the GyrB TOPRIM domain, re‐ligation fails, stalling the catalytic cycle and trapping the enzyme‐DNA complex in an inactive state.

Importantly, corramycin's activity is dependent on the presence of ATP, suggesting that ATP binding promotes the essential conformational changes required for binding of the compound. Supporting this, the identification of resistance‐conferring mutations within the GyrA CTD (G694) and in the disordered linker connecting the BRD to the CTD (L510, Figure 3a,b), distant from the corramycin binding site, is consistent with drug binding being intimately linked to the conformation of gyrase and the state of the dsDNA. It is well‐established that the GyrA CTD is essential for the supercoiling activity of the enzyme, as it wraps the DNA around itself [52, 53]. Thus, we hypothesize that the observed substitutions alter the properties of the CTDs, impacting DNA binding and, consequently, modifying the conformation of gyrase. These conformational changes may ultimately inhibit corramycin binding, revealing an allosteric resistance mechanism. This observation aligns with the lack of activity against topo IV (Figure 4c). Although the catalytic cores of gyrase and topo IV are highly conserved, their CTDs differ substantially, and it was shown that these domain‐specific differences contribute to the discrimination of activity between the two enzymes [54, 55].

Finally, corramycin is known to be inactivated in its native host via phosphorylation of the *β*‐hydroxyl group of the hydroxylated *γ*‐amino valeroyl moiety by the ComG kinase [10]. In the corramycin‐bound structure, the phosphorylation site is located near D461 of the GyrB subunit, offering insight into how the introduction of a negatively charged phosphate group could interfere with binding by causing an electrostatic clash with the adjacent aspartate (Figure 6a).

The specific antibacterial activity of corramycin against *Mtb* and *E. coli*, but not Gram‐positive pathogens, raises the question of whether this narrow antibacterial spectrum can be explained at the molecular level. However, the selective activity cannot be reasoned on the basis of homology between bacterial gyrase enzymes. In fact, structural comparisons reveal that the binding pockets of the *Mtb*, *E. coli* and *S. aureus* gyrases are nearly identical (Figure S18). This is in line with in vitro assays, in which corramycin induces DNA cleavage mediated by *S. aureus* gyrase to an extent similar to that with the *E. coli* gyrase (Figure S10). Instead, the selective activity of corramycin appears to result from its reliance on specific transport system such as BacA and its homolog SbmA [9]. Interestingly, BacA/SbmA‐dependent uptake has also been observed for the DNA gyrase inhibitor evybactin, which displays a spectrum of activity similar to that of corramycin [44]. Apart from being a peptidic molecule, evybactin shares no apparent chemical similarity with corramycin, suggesting that BacA/SbmA‐like importers might function as promiscuous transporters capable of facilitating the uptake of a wide range of structurally diverse peptidic drugs. In addition to BacA, we identified the OppABCD transport system in *Mtb* as another likely entry route for corramycin. In *E. coli*, corramycin has also been shown to benefit from transporter redundancy [9]. The utilization of multiple importers has the advantage that resistance development is likely slow as it would require concurrent mutation events in all involved uptake systems.

Corramycin overcomes resistance associated with first‐, second‐ and last‐line drugs, including FQs, yet still exhibits the DNA damaging activity that is required for DNA gyrase‐targeting compounds to kill non‐replicating, caseum‐adapted *Mtb* [56], positioning this compound class as an intriguing starting point for developing a novel antibiotic drug candidate. Due to limited knowledge of corramycin's MOA, past optimization efforts have largely focused on eliminating instabilities and improving bacterial uptake [11]. Our work now paves the way for rational, structure‐based drug design to guide the development of next‐generation corramycins featuring optimized scaffolds. Notably, the *γ*‐*N*‐methyl‐*β*‐OH‐histidine proved crucial for binding to gyrase. However, this key moiety has also proven to be a major bottleneck in the total synthesis, hampering the production of large quantities [57]. Future efforts will therefore focus on the substitution of this group to facilitate scalable synthesis and analog development. In parallel, further optimization will be required to better align the corramycin scaffold with the target product profile for TB, particularly with respect to intracellular activity and pharmacokinetic PK.

## 4 Methods

### 4.1 Ethics Statement

The Ethics Committee of the University of Lübeck approved the isolation of and experimentation with human peripheral blood cells (AZ2023‐450_1). Written consent approving and authorizing the use of the volunteers’ material for research purposes was obtained from all donors.

### 4.2 Biosafety Statement

All experiments with virulent *Mtb* strains were conducted in authorized BSL‐3 laboratories by trained personnel in accordance with applicable biosafety regulations.

### 4.3 Minimum Inhibitory Concentration

Corramycin stock solutions were prepared in dimethyl sulfoxide (DMSO). All microorganisms used in this study were obtained from the German Collection of Microorganisms and Cell Cultures (DSMZ), the American Type Culture Collection (ATCC), the Coli Genetic Stock Center, or were part of our internal strain collection. *S. aureus* Newman was obtained from M. Bischoff, Saarland University Hospital, Homburg. *E. coli* WO153 was provided by K. Lewis, Northeastern University, Boston, USA.

Minimum inhibitory concentrations (MICs) were determined using the broth microdilution method according to EUCAST guidelines (ISO 20776‐1:2019). In short, serial two‐fold dilutions of corramycin (0.03125 to 64 µM) were prepared in 75 µL of cation‐adjusted Mueller‐Hinton broth (MHB2) in sterile 96‐well plates. Equal volume of the bacterial suspension was added, and the plates were incubated at 37°C for 18 h. For *Streptococcus pneumoniae*, MHF broth (MHB2 supplemented with 5% lysed horse blood and 20 mg/L β‐NAD) was used and plates were incubated at 37°C with 5% CO_2_. The MIC was defined as the lowest concentration of the antibiotic causing complete inhibition of visible growth of the microorganism. The same method was used for testing *M. smegmatis*, but with the use of Middlebrook 7H9 complete medium supplemented with oleic acid, albumin, dextrose and catalase (OADC, 10%). *M. smegmatis* plates were incubated for 48 h at 37°C.

For assessing activity against *Mtb* H37Ra, *M. avium* (ATCC700898, MAC101), *M. intracellulare* (ATCC13950) and *M. abscessus* (ATCC19977), an adapted resazurin microtiter assay (REMA) was performed. In short, single cell suspensions of *Mtb* H37Ra (2 × 10^5^ CFU/mL) and nontuberculous mycobacteria (1 × 10^5^ CFU/mL) were prepared and added to compound dilutions in 7H9 supplemented with 10% OADC. Plates were incubated for 6 days (*Mtb*, *M. intracellulare*), 3 days (*M. avium*) or 1 day (*M. abscessus*) at 37°C, followed by addition of 50 µL of 0.01% (*w/v*) resazurin and incubation for another day at 37°C. The MIC was determined visually and additionally confirmed by measuring fluorescence using a plate reader (Tecan, Spark) (ex/em: 540/590 nm).

*Mtb Erdman* (ATCC 35801), including spontaneous resistant mutants and recombineered strains as well as moxifloxacin‐resistant mutants (H37Rv background) were tested using a previously described REMA assay [58]. The strains were cultured in 7H9 broth supplemented with 10% (*v/v*) ADC, 0.2% (*v/v*) glycerol and 0.05% (*v/v*) Tween‐80 (7H9c) to an OD_560_ of 1.0. Frozen aliquots containing 25% glycerol were prepared. For testing, a frozen vial was thawed and diluted in 7H9c to an OD_560_ of 0.0003. 90 µL of the bacterial suspension was added to compounds prediluted 1:2 in sterile ddH_2_O. Plates were sealed with adhesive film and incubated at 37°C for 7 days. 10 µL of 0.025% resazurin was added, and plates were incubated for an additional 24 h before fluorescence was measured as described above. Data were normalized to 0.1% DMSO (0%) and 5 µM rifampicin (100%) controls. MIC_95_ values were determined by fitting the data to a 4‐parameter log‐normal model in R and the drc package [59, 60].

*Mtb* H37Rv (ATCC 27294) as well as DS *Mtb* clinical isolates were cultured in 7H9 complete medium supplemented with 10% OADC, 0.2% (*v/v*) glycerol and 0.05% tyloxapol (*v/v*) until an OD_600_ of 0.4. Prior to testing, cultures were centrifuged (1,900 *g*, 10 min). Supernatants were discarded, bacteria washed twice with PBS, and subsequently thoroughly resuspended (5 times) in 7H9 (10% OADC) by use of a syringe and a 26‐gauge syringe needle. Compound efficacy was assessed using a broth microdilution assay, based on a previously described protocol with a few modifications [61]. Briefly, two‐fold serial dilutions of each compound were prepared starting at a concentration of 64 µM. Wells were inoculated with *Mtb* (1 × 10^5^ CFU) in a final volume of 100 µL, using clear, U‐bottom 96‐well microtiter plates to evaluate antitubercular activity. Plates were left to incubate at 37°C, in ziplock bags within humidified sealed boxes for 7 days prior to visual examination of bacterial growth using an inverted mirror device. 30 µL of a 0.02% (*w/v*) aqueous solution of resazurin (Cayman Chemical, Ann Arbor, USA) was then added to each well and the plates left to incubate at 37°C for 4 h. Reduction of resazurin was measured with a fluorescence plate reader as described above. Fluorescence values were converted to percentage growth relative to DMSO treated cells (100% growth). Final DMSO concentrations did not exceed 1% (*v/v*). The obtained values were normalized to fluorescence values of the solvent control (DMSO‐treated bacteria set to 100%), and MIC_95_ of each compound was determined. MIC_95_ was defined as the minimum concentration of the compound required to achieve a reduction in fluorescence by 95%.

MIC testing of DR *Mtb* isolates was performed according to the EUCAST protocol (Version 8.2, EUCAST AntiMycobacterial Susceptibility Testing Subcommittee, 2025). Briefly, *Mtb* inocula were prepared from a 1:100 dilution of a 0.5 McFarland suspension, corresponding to a CFU/mL of 1 × 10^5^. For the 1% growth control, the suspension was additionally diluted 1:100. The microtiter plates were inoculated with 100 µL of *Mtb* suspensions. On days 7, 10, 14, and 21 the plates were examined. With first visual growth of the 1% control results were recorded. The MIC corresponds to the lowest concentration of the substance at which no visible growth is observed.

### 4.4 Cytotoxicity

Human A‐549 lung carcinoma (ACC 107), Chinese hamster ovary CHO‐K1 (ACC 110), human HEK‐293 embryonal kidney (ACC 305), and human HepG2 hepatoblastoma cells (ACC 180) were obtained from the German Collection of Microorganisms and Cell Cultures (DSMZ) and cultured under the conditions recommended by the depositor. Cells were propagated in Dulbecco's Modified Eagle Medium (DMEM) (A‐549, HEK‐293 and HepG2) or Ham's F12 medium (CHO‐K1) supplemented with 10% fetal bovine serum (FBS), and seeded at 6 × 10^3^ cells per well of 96‐well plates in 120 µL of complete medium. After 2 h of equilibration (37°C, 5% CO_2_), the cells were treated with a serial dilution of corramycin. Corramycin, doxorubicin as reference, as well as the solvent control (DMSO) were tested as duplicates in two independent experiments. After 5 d of incubation (37°C, 5% CO_2_), a total of 20 µL of 5 mg/mL MTT (thiazolyl blue tetrazolium bromide) in phosphate‐buffered saline (PBS) were added to each well and the cells were further incubated for 2 h at 37°C before the supernatant was discarded. Subsequently, the cells were washed with 100 µL of PBS and treated with 100 µL of 2‐propanol/10 N HCl (250:1) to dissolve formazan granules. Cell viability was measured as a percentage relative to the respective solvent control by measuring the absorbance at 570 nm using a microplate reader (Tecan Infinite M200Pro). GraphPad Prism (version 10.0.3, GraphPad, Boston, MA, USA) was used for sigmoidal curve fitting to determine the IC_50_ values.

### 4.5 Intracellular Activity in Human Primary Macrophages

Human monocyte‐derived macrophages (hMDMs) were generated as described [62]. hMDMs (2 × 10^5^) were cultured in 500 µL RPMI 1640 with 10% FCS and 4 mM l‐glutamine in 48‐well flat‐bottom microtiter plates (Nunc) at 37°C in a humidified atmosphere containing 5% CO_2_. Macrophages were infected with *Mtb* H37Rv at a multiplicity of infection of 0.5:1 mycobacteria/macrophage for 4 h [62]. Subsequently, non‐phagocytosed bacteria were removed by washing three times with 0.5 mL Hanks’ balanced salt solution (HBSS) (PAN Biotech, Aidenbach, Germany) at 37°C. Cells were then cultured in 0.5 mL culture medium in the absence or presence of corramycin or rifampicin (in DMSO) for 7 days (37°C, 5% CO_2_). DMSO (0.1%) was used as a solvent control. On day 3, 0.5 mL culture medium was added to the macrophage culture. At day 7, supernatants were completely removed, and macrophage cultures were lysed by adding 0.05% Tween‐80/dH_2_O at 37°C for 15 min. Lysates were serially diluted in sterile water containing 0.05% Tween‐80 (Merck, Darmstadt, Germany) and plated twice on 7H10 agar containing 0.5% glycerol (Carl Roth, Karlsruhe, Germany) and 10% heat‐inactivated bovine calf serum (BioWest, France). After 3 weeks at 37°C, the CFU counts were determined.

### 4.6 Time‐Kill Kinetics

A liquid culture of *Mtb* H37Ra was grown in growth medium (supplemented with 0.4% (*v/v*) glycerol and 0.05% (*v/v*) Tween‐80) until log‐phase. Single cells were prepared by syringing and the count was adjusted with growth medium (without glycerol and without Tween‐80) to reach 10^6^ CFU/mL. The culture was split into 5 mL samples and supplemented with different concentrations of corramycin, 10x MIC of moxifloxacin (MIC *Mtb* H37Ra 0.125 µg/mL) or DMSO (control). Cultures were incubated at 37°C, 180 rpm. At designated time points, an aliquot of the samples was taken and appropriate dilutions were plated on M7H11 agar supplemented with 10% OADC. The plates were incubated at 37°C for five weeks and bacterial counts were determined.

For *Mtb* Erdman, a pre‐culture was grown to an OD_560_ of 1 and subsequently diluted to an OD_560_ of 0.003 (approx. 1 × 10^6^ CFU/mL). Aliquots of 10 mL were transferred into 30 mL square bottles (Thermo Scientific), and the respective compounds were added. DMSO (< 1%) served as control. Cultures were incubated statically at 37°C with 5% CO_2_. On days 0, 2, 4, 7, 10, 14, 21, 30, and 50, the cultures were mixed, and three samples of 100 µL were collected from each bottle. Ten‐fold serial dilutions were prepared in PBS, and 10 µL of each dilution was spotted onto 7H10 plates. Plates were incubated at 37°C for 21 days, after which CFUs were enumerated.

### 4.7 Synergy Testing

Synergy of antibiotics was evaluated using the checkerboard assay. Two‐fold dilutions of corramycin (COR; plate 1) and dilutions of the combination antibiotic (AB) were prepared (plate 2) in 7H9 complete medium supplemented with 10% OADC. In plate 1, corramycin was serially diluted along the y‐axis, whereas in plate 2, a serial dilution of the combination antibiotic was prepared along the x‐axis. 50 µL from wells B1‐H11 of plate 1 were transferred to the corresponding wells of plate 2, resulting in a checkerboard pattern. A single cell suspension of *Mtb* H37Ra with a bacterial count of 3 × 10^5^ CFU/mL was prepared and 50 µL of this bacterial suspension was dispensed into each well of plate 2 apart from well H12 (negative control). The plate was incubated for 6 d at 37°C. MICs of antibiotics (AB) alone as well as of each combination were determined using resazurin as described above. In order to quantify the interactions between the two antibiotics, the Fractional Inhibitory Concentration Index (FICI) was calculated using the following equation:

$$\sum \mathrm{FICI}=\mathrm{FI}C_{\mathrm{COR}}+\mathrm{FI}C_{\mathrm{AB}}=\frac{\mathrm{MI}C_{\mathrm{COR}/\mathrm{combi}}}{\mathrm{MI}C_{\mathrm{COR}}}+\frac{\mathrm{MI}C_{\mathrm{AB}/\mathrm{combi}}}{\mathrm{MI}C_{\mathrm{AB}}}$$

A FICI ≤ 0.5 showed synergism between the two antibiotics, a FICI between 0.5 and 1 showed an additive effect, values between 1 and 2 were considered as indifferent and a FICI ≥ 2 indicated antagonism [63]. Checkerboards assays were performed in two independent biological repeats.

### 4.8 *M. tuberculosis* Reporter Strain Assay

Reporter constructs were generated through standard molecular biology techniques, using the Mycobacteriophage Giles site integrating plasmid pGiles [64] (a gift from Clifton E. Barry III, NIH, Bethesda, MD, USA) as the backbone. Briefly, the hygromycin resistance gene of pGiles was first replaced with a nourseothricin resistance gene (derived from plasmid pGMCtN‐TSC10M1‐sspB, Addgene plasmid #49521, a gift from Dirk Schnappinger, Weill Cornell Medicine, NY, USA) [65], followed by insertion of an open reading frame coding for mNeonGreen (derived from plasmid pDC‐UAS‐nBitbow1.0(miniW), Addgene plasmid #138329, a gift from Dawen Cai, University of Michigan, MI, USA) [66] downstream of the pSmyc promoter (between restriction sites NcoI and HindIII). Next, a ∼230 bp DNA fragment derived from a self‐designed synthetic gene (Eurofins Genomics, Ebersberg, Germany) was PCR amplified using primers incorporating NotI (5’) and BamHI (3’) restriction sites and inserted into the NotI and BglII sites of the resulting plasmid. The combination of compatible BglII and BamHI sites at this point inactivated the original BglII site, allowing reintroduction of a new BglII site at a different location. This DNA fragment introduced into the plasmid (1) a BglII site directly downstream of the NotI site; (2) a short 40 bp linker sequence derived from the promoter region of Rv0440 (nucleotide ‐59 to ‐19 relative to the start codon; does not contain any functional promoter elements); (3) a strong ribosome binding site (AGGAGG) followed by a short 6 bp linker sequence; (4) a FLAG‐tag encoding sequence commencing with an ATG start codon; (5) NdeI (in‐frame with FLAG tag) and BstBI restriction sites; (6) a transcriptional terminator. This fragment allows cloning of secondary reporter genes between the NdeI and BstBI sites, and test promoters into the NotI and BglII sites. In this way, an open reading frame coding for stagRFP (derived from plasmid pcDNA3‐StagRFP, Addgene plasmid #173014, a gift from Jin Zhang, University of California, San Diego, CA, USA) [67] was PCR amplified and cloned into the NdeI and BstBI sites, followed by introduction of desired mycobacterial promoter regions between the NotI and BglII sites. Promoter regions were chosen based on previous publications, and were defined as DNA sequences commencing directly upstream of the putative ribosome‐binding site of the candidate genes, and continuing upstream for 200–500 bp. The chosen promoters (identified by their corresponding gene) and lengths were as follows: Rv0341 (*iniB)* [18]: 215 nt; Rv1623 (*cydA*) [19]: 280 nt; Rv2383 (*mbtB)* [17]: 190 nt; Rv2737 (*recA)* [18]: 461 nt.

Resulting plasmids were electroporated into *Mtb* H37Rv along with the Giles‐integrase encoding suicide plasmid pUC19 Giles integrase (Addgene plasmid #170049, a gift from Clifton E. Barry III, NIH, Bethesda, MD, USA) [64] and positive transformants selected on nourseothricin (25 µg/mL) containing solid media (Middlebrook 7H10 agar supplemented with 10% OADC). Strains were grown to mid‐exponential phase in liquid 7H9 supplemented with 5 g/L bovine serum albumin, 4 g/L glucose, 0.85 g/L NaCl, and 0.5 g/L tyloxapol, 0.1 mM sodium propionate and 25 µg/mL nourseothricin at 37°C, 100 rpm. Reporter assays were performed in U‐bottom, clear 96‐well plates with a final volume of 100 µL/well, using the same media described above. Compounds were added in an 11‐point, 2‐fold dilution series (maximum DMSO concentration of 0.1% (*v/v)* for the highest compound concentrations) across plate rows. Untreated and 5 µM rifampicin treated wells were included on each plate as 100% and 0% (respectively) growth controls. Bacteria were added to a starting OD_600_ of 0.1 (∼5 × 10^5^ cells/well) and plates were incubated statically at 37°C in sealed, humidified boxes. Fluorescence measurements were recorded at desired time points using a Synergy2 Multi‐mode microplate reader (Agilent), employing filter sets 485/20 nm (excitation) and 528/20 nm (emission) to monitor mNeonGreen expression, and 540/35 nm (excitation) and 590/20 nm (emission) for stagRFP expression. Experiments were performed in two independent biological replicates.

### 4.9 Transcriptome Analysis

A log‐phase culture of *E. coli* ATCC25922 in MHB2 was diluted to an OD_600_ of 0.5. Bacteria were subsequently treated with different concentrations of corramycin (0.125x, 0.25x and 0.5x MIC, respectively) for either 15 min or 45 min. For the untreated control, the same volume of DMSO was added. Each condition was performed in triplicates. At the designated time points, 500 µL of samples was centrifuged at 5,000 *g*, RT, 10 min. The supernatant was removed and pellets were dissolved in TE buffer (100 mM Tris, 1 mM EDTA, pH 8.0) containing 15 mg/mL lysozyme and 20 µL of proteinase K (Qiagen). Samples were mechanically disrupted with acid‐washed glass beads (one spatula tip of sizes 150–212, 212–300, and 425–600 µm, respectively) using the TissueLyser II (Qiagen) at a frequency of 1/s for 5 min. 700 µL QIAzol lysis reagent (Qiagen) was added and samples were disrupted for another two cycles with the TissueLyser II. Subsequently, total RNA was extracted using the RNeasy mini kit (Qiagen).

For *Mtb* treatment with corramycin, single cells of a log‐phase culture were prepared. The OD_600_ was adjusted to 0.5 with growth medium and bacteria were subsequently exposed to different concentrations of corramycin (0.5x, 1x and 2x MIC, respectively) for either 6 or 24 h. For the untreated control, the same volume of DMSO was added. Each condition was performed in triplicates. At the designated time points, 7 mL of samples was drawn and total RNA was extracted following a protocol recently described, consisting of mechanical disruption with the FastPrep machine (MP Biomedicals, Irvine, CA, USA) and extraction using the RNeasy mini kit (Qiagen) [68].

Concentration and quality of the RNA were determined using the NanoDrop (Thermo Fisher Scientific, Waltham, MA, USA) and Agilent 2100 Bioanalyzer (Santa Clara, CA, USA). Sequencing of *E. coli* RNA was performed on the NovaSeq 6000 in cooperation with the Genome Analytics group at the Helmholtz Centre for Infection Research (HZI, Braunschweig, Germany). Sequencing of *Mtb* RNA was performed using Illumina technology at Azenta Life Sciences (Burlington, MA, USA). Raw sequencing data was analyzed using Geneious Prime (version 2023.1.1). Reads were mapped to the reference sequence (*E. coli* ATCC25922 NCBI NZ_CP009072.1; *Mtb* H37Rv NCBI NC_000962.3) prior to normalization and differential gene expression analysis with DESeq2. *P*‐values were adjusted for multiple testing using the Benjamini‐Hochberg procedure for false discovery rate (FDR) correction.

### 4.10 Proteome Analysis

For *E. coli* ATCC25922, a liquid overnight culture was inoculated (1:100) in 5 mL fresh LB medium and treated with 0.125x MIC of corramycin, or the respective amount of DMSO as negative control. The liquid cultures were incubated at 37°C, 180 rpm until they reached early stationary growth phase (*n* = 4). Subsequently, 1 mL sample was collected in an Eppendorf tube on ice, centrifuged (6,000 *g*, 4°C, 5 min) and washed with 1 mL cold PBS, before storing the pellet at ‐80°C until further use. *Mtb* H37Ra was grown until early log‐phase (OD_600_ 0.4) and diluted to an OD_600_ of 0.2 in 5 mL of fresh 7H9 medium (supplemented with 10% OADC) and treated with 0.125x MIC of corramycin, or the respective amount of DMSO as negative control. The liquid cultures were incubated at 37°C, 180 rpm for 5 d (*n* = 4). Subsequently, 5 mL was transferred into a 15 mL falcon tube on ice, centrifuged (6,000 *g*, 4°C, 5 min), re‐suspended in 1 mL of cold PBS and transferred into a 1.5 mL centrifuge tube. The sample was centrifuged again and the pellet was stored at ‐80°C until further use.

Lysis was carried out by addition of 210 µL of 0.4% SDS in PBS and three rounds of sonication with an amplitude of 50% for 30 s (Bandelin Sonoplus, Berlin, Germany). Subsequently, samples were centrifuged (16,900 *g*, 20 min, RT) and the supernatant was used to determine the protein concentration using a BCA assay (Thermo). Proteome concentrations of the samples were adjusted to 100 µg/200 µL per sample. Proteome was precipitated by addition of 1 mL ice‐cold acetone and incubation overnight at ‐20°C. Next, the samples were centrifuged (16,900 *g*, 15 min, 4°C) and the supernatant was discarded. Samples were washed twice with 1 mL of ice‐cold methanol. Protein digest was done by resuspension in 200 µL X‐buffer (7 M urea, 2 M thiourea, 20 mM HEPES, pH 7.5) followed by reduction of cysteine with 0.8 µL of 250 mM DTT (dithiothreitol) for 45 min at 25°C, capping with 2 µL 550 mM IAA (iodoacetic acid) for 30 min at 25°C and addition of additional 3.2 µL 250 mM DTT for 30 min at 25°C. 600 µL ammonium bicarbonate buffer (pH 8.5) and 1 µg of trypsin (MS grade, Promega, Madison, WI, USA) were added for overnight digest at 37°C, 400 rpm shaking. Digestion was stopped by addition of 8 µL formic acid (FA). Peptide samples were desalted on SepPak C18 columns (Waters, Milford, MA, USA) using the following protocol. Columns were primed by adding 1 mL of 100% acetonitrile (ACN) and 0.5 mL of 80% ACN with 0.5% FA, respectively, followed by equilibration with 3 × 1 mL of 0.1% TFA, before samples were loaded and washed three times with 1 mL of 0.1% TFA and once with 0.5 mL of 0.5% FA. Subsequently, samples were eluted into 2 mL LoBind tubes (Eppendorf, Hamburg, Germany) by adding 3 × 250 µL of 80% ACN + 0.5% FA under slight vacuum, and dried using the Eppendorf Concentrator Plus. Dried samples were then solved in 1% FA to a final proteome concentration of 1 µg/µL. Samples were filtered through a 0.22 µm membrane (Merck Millipore, UFC30GVNB) and transferred to QuanRecovery autosampler vials (Waters). Sample analysis was done by using nanoElute nano flow liquid chromatography system (Bruker, Germany) coupled with a timsTOF Pro (Bruker, Germany). Samples were loaded to the trap column (Thermo Trap Cartridge 5 mm) and washed with 6 µL 0.1% FA with a flow rate of 10 µL/min. Peptides were then transferred to the analytical column (Aurora Ultimate CSI 25 cm x 75 µm ID, 1.6 µm FSC C18, IonOpticks) and separated by a gradient elution (eluent A: H_2_O + 0.1% FA, B: ACN + 0.1% FA; 0% to 3% in 1 min, 3% to 17% in 57 min, 17% to 25% in 21 min, 25% to 34% in 13 min, 34% to 85% in 1 min, 85% kept for 8 min) with a flow rate of 400 nL/min. Captive Spray nanoESI source (Bruker, Germany) was used to ionize the peptides at 1.5 kV with 180°C dry temperature at 3 L/min gas flow. TimsTOF Pro (Bruker, Germany) was operated in default dia‐PASEF long gradient mode with TIMS set to 1/K0 start at 0.6 Vs/cm^2^, end at 1.6 Vs/cm^2^ with a ramp and accumulation time of 100 ms each and a ramp rate of 9.43 Hz. Mass range was set from 100.0 Da to 1700 Da with positive ion polarity. Dia‐PASEF mass range was set to 400.0 Da to 1201.0 Da with a mobility range of 0.60 1/K0 to 1.43 1/K0 and a cycle time of 1.80 s. Collision energy for 0.60 1/K0 was set to 20.00 eV and for 1.6 1/K0 to 59.00 eV. Tuning MIX ESI‐TOF (Agilent) was used for calibration of *m/z* and mobility. Data were processed using DIA‐NN (version 1.8.1), and proteins were identified using Uniprot *E. coli* K12 reference proteome (Proteome ID: UP000000625, downloaded 13/04/2023) or *Mtb* H37Rv (Proteome ID: UP000001584, downloaded 17/12/2022). Settings were used as default except precursor charge range was from 2 to 4. C carbamidomethylation was set as fixed modification. “–relaxed‐prot‐inf” was added in additional options to allow further data processing with Perseus Software. In Perseus (version 2.0.7.0) the values were transformed to their log_2_‐value and the replicates were grouped. The results were filtered by valid values in 3 out of 4 in at least one group. Missing values were imputed by default settings and the differential protein abundances between different conditions were evaluated using two‐tailed student's T‐test. Cut‐off for –log_10_ *p*‐value was set to 1.3 (*p*‐value 0.05) and T‐test difference > 1.5. Proteins fitting these thresholds were identified as significantly over‐ or under‐expressed compared to the controls.

### 4.11 Scanning Electron Microscopy

An overnight culture of *E. coli* ATCC25922 was sub‐cultured 1:50 in fresh medium and re‐incubated until mid‐log phase was reached. The culture was divided in 2 mL samples and exposed to 2x or 4x MIC of corramycin, or DMSO (control). Samples were incubated for 2 h at 37°C. Cells were fixed by incubating 1440 µL of the sample with 160 µL of 25% glutaraldehyde for 30 min at room temperature, followed by addition of 400 µL of 25% paraformaldehyde and incubation for another 30 min. For *Mtb*, cells were grown in 7H9 supplemented with 10% OADC and 0.05% (*v/v*) Tween‐80 until log‐phase. Single cells were prepared and the OD_600_ was adjusted to 0.2. The bacterial suspension was divided in 5 mL samples exposed to 5x MIC of corramycin, or DMSO (control), and incubated for 3 days at 37°C. Fixation was performed by addition of 160 µL of 25% glutaraldehyde and 400 µL of 25% paraformaldehyde to 1440 µL of the samples, and incubation for 30 min at room temperature. Samples for SEM were processed as previously described [69]. In brief, bacteria were fixed to 12 mm, round, poly‐l‐lysine pre‐treated cover slips and dehydrated in a graded series of acetone on ice, critical‐point dried with liquid CO_2_ (CPD 300, Leica Microsystems, Wetzlar, Germany) and sputter coated with a gold‐palladium film (SCD 500, Bal‐Tec, Lichtenstein). Image acquisition was performed with a field‐emission scanning‐electron microscope Zeiss Merlin (Oberkochen, Germany) using Everhart Thornley and the inlens SE detectors at an acceleration voltage of 5 kV. Cellular length of *Mtb* was measured manually using Fiji ImageJ software (version 2.3.0). A minimum of 200 cells (exact numbers are given in the plots) were measured per condition.

### 4.12 DNA Displacement Assay

20 µM of calf thymus (CT‐)DNA (Sigma‐Aldrich, St. Louis, MO, USA) along with 15 µM of Hoechst33342 (Sigma‐Aldrich) or 10 µM of ethidium bromide (Sigma‐Aldrich) were incubated with different concentrations of corramycin, doxorubicin (Cayman Chemical, Ann Arbor, MI, USA) or netropsin (Sigma‐Aldrich) in the dark for 30 min at room temperature. For Hoechst33342 displacement, emission spectra were recorded from 390 to 850 nm at an excitation wavelength of 355 nm. For ethidium bromide displacement, emission spectra were recorded from 530 to 850 nm at an excitation wavelength of 480 nm. Fluorescence intensity from inhibitor with CT‐DNA alone was also measured and used for subsequent subtraction in the final graph.

### 4.13 Resistance Studies

To obtain spontaneous resistant mutants, *Mtb* Erdman was cultured to an OD_560_ of 1.0. Up to 10 mL of bacterial suspension was centrifuged at 8,000 *g* for 5 min, the supernatant discarded, and the resulting pellet resuspended in 100 µL of liquid broth. The suspension was then plated onto 7H10 agar supplemented with 10% OADC, 0.5% glycerol and corramycin at concentrations ranging from 2 to 50 µM, depending on the selection stage. For the selection of mutants in liquid culture, 5 µM corramycin was added to 7H9c broth in a culture flask, and bacteria were inoculated to an OD_560_ of 0.05. The culture was incubated under static conditions at 37°C. Once the OD_560_ reached 1, a separation smear was performed. Putative resistant colonies were isolated and sub‐cultured in 7H9c medium without corramycin. Resistance was confirmed using the REMA assay, and validated resistant clones were then extracted using a cetyltrimethylammonium bromide (CTAB)‐based extraction method. Sequencing libraries were prepared using the Celero DNA‐Seq Library Prep kit (Tecan Trading AG, Switzerland) and subjected to 2 × 151 bp sequencing on Illumina's NextSeq 2000 machine (minimum 50x coverage).

For the serial passaging approach, single cells of a log‐phase *Mtb* H37Ra culture were prepared by syringing and subsequently, the suspension was diluted to reach an OD_600_ of 0.05. The suspension was split into 11 cultures, 10 of which were supplemented with 0.5x MIC of corramycin (0.5 µM). The remaining culture was supplemented with the same volume of DMSO (wild‐type). All cultures were incubated at 37°C. When the samples turned turbid, they were sub‐cultured 1:50 in fresh growth medium (7H9 + 10% OADC) with a 50% increased corramycin concentration. This process was repeated until the corramycin concentration reached 8.4 µM. The putative mutant cultures were tested for antimicrobial susceptibility as described above. Isolation of gDNA from *Mtb* was performed using an adapted CTAB method [68]. gDNA of *Mtb* H37Ra resistant mutants and the respective wild‐types were sequenced using Illumina Paired‐End technology on the MiSeq instrument (2 × 300 bp, 100x coverage) in cooperation with the Genome Analytics group at the Helmholtz Centre for Infection Research (HZI, Braunschweig, Germany).

Mutants harboring variants in *gyrA* (D89N, A90V, D94G/H/N) or *gyrB* (N499K) were generated as previously described [70]. Briefly, *Mtb* H37Rv was cultured in 7H9 supplemented with 10% OADC, 0.2% glycerol, and 0.05% Tween‐80 at a starting OD_600_ of 0.05 and exposed to 0.5x, 0.25x, or 0.125x MIC of moxifloxacin (MOX) (MIC 0.12 mg/L). Cultures were plated on 7H10 supplemented with 10% OADC, 0.5% glycerol, and 0.25 µg/mL MOX. Single mutant colonies were isolated and re‐grown in MOX‐free medium. DNA extraction and WGS was performed as described for corramycin‐resistant *Mtb* Erdman mutants. The mutants selected for this study were passaged one time.

Raw sequencing data were analyzed using the MTBseq v1.1.0 pipeline with standard parameters [71]. In short, reads were mapped to the *Mtb* reference genome (*Mtb* H37Rv NCBI NC_000962.3), and variants were identified with samtools pileup and individual filter criteria. In the used default mode, a variant was called if it was supported by a minimum number of 4 reads in both forward and reverse direction, by a minimum number of 4 reads indicating the allele with a phred score of at least 20, and if the allele was indicated by a minimum frequency of 75% in mapped reads. For comparison of mutants and reference, the sequences were combined with the *TBjoin* command in MTBseq. All identified mutations are summarized in Tables S3 and S5. The FQ‐resistant mutants had no additional variants outside the *gyrA* or *gyrB* variants described.

### 4.14 Site‐Directed Mutagenesis of Gyrase Genes

*Mtb* Erdman harboring the plasmid pVJ53 [27] was grown to mid‐log phase in 7H9c broth containing 25 µg/mL kanamycin and induced with 0.2% acetamide and 0.2 M glycine for 24 h. Electrocompetent cells were prepared via multiple washing steps with 10% glycerol and transformed with 100 ng of single‐stranded oligonucleotides of leading and lagging strands (Table S9). Transformants were selected on 7H10 agar plates supplemented with 10% OADC, 0.5% glycerol and 5 µM corramycin, and resistant clones were confirmed by WGS. The recombineered mutants had no additional variants outside the introduced *gyrA* or *gyrB* variants.

### 4.15 Profiling of Corramycin by CRISPRi

CRISPRi profiling was performed as described previously [72, 73]. Briefly, triplicate cultures of the CRISPR library RLC0025 [73] supplemented with anhydrotetracycline (ATc, 100 ng/mL), kanamycin (10 µg/mL), and DMSO or corramycin dissolved in DMSO were prepared. Cultures were grown for 14 days at 37°C, 5% CO_2_ and ATc was replenished at day 7. Genomic DNA was isolated from bacterial pellets using the Omega Bio‐Tek Mag‐Bind Universal Pathogen DNA Kit (M4029), along with a Geno/Grinder for cell lysis and a Hamilton Starlet for partial automation of the extraction process. The concentration of isolated genomic DNA was quantified using a Thermo Scientific NanoDrop ND‐8000 Spectrophotometer. Subsequently, the sgRNA‐encoding region was amplified, purified, pooled for Illumina sequencing, and analyzed using MAGeCK as previously described [72].

### 4.16 Protein Production and Purification

Expression and purification of the *Mtb* fusion‐gyrase GyrBA (consisting of GyrB fused to GyrA through a three‐amino acid G‐D‐L linker) were performed as previously described [35, 49]. In brief, *E. coli* BLi5 (DE3) [74] was transformed with pET‐28a constructs harboring the ORF of interest, and expression of N‐terminally His‐tagged proteins was induced via a lactose‐driven autoinduction. Following overnight growth at 30°C, bacteria were harvested by centrifugation and resuspended in buffer A (20 mM Tris‐HCl pH 8, 500 mM NaCl, 10 mM imidazole, 1 mM DTT, 5 mM MgCl_2_, 5% Glycerol) supplemented with a cOmplete ULTRA EDTA‐free protease inhibitor tablet (Roche Diagnostics). Bacterial lysis was conducted on a Cell Disruption Lysis System. Proteins were then purified from the soluble extract on a Ni^2+^ affinity column (HisTrap HP column, Cytiva) with a linear imidazole gradient. Fractions of interest were subjected to TEV digestion in a dialysis buffer (10 mM Tris‐HCl pH 7.5, 80 mM NaCl, 1 mM DTT, 1mM EDTA and 10% glycerol). Untagged proteins were then collected after a passage on a Heparin column (Heparin HP column, Cytiva) with a linear NaCl gradient (80 to 1000 mM) and a size‐exclusion chromatography (Superdex 200 column, Cytiva) in buffer B (20 mM Tris‐HCl pH 7.5, 100 mM KCl, 1 mM DTT, 1 mM EDTA, 10% glycerol). Purified proteins were flash‐frozen in liquid nitrogen and stored at −80°C. Protein concentration was determined using the molar absorption coefficient predicted from the amino acid sequence by the ProtParam tool [75].

### 4.17 Topoisomerase Assays

All topoisomerase assays were performed according to the manufacturer's protocol (Inspiralis, Norwich, UK). Wild‐type enzymes are the native A_2_B_2_ assemblies supplied with the Inspiralis gyrase assay kits. Resistant enzymes were specifically engineered by Inspiralis (*Mtb* GyrA D89E – GyrB WT and GyrA WT – GyrB R482L). For DNA gyrase cleavage assays, 30 µL reactions were set up, consisting of assay buffer, 1 mM ATP, 0.5 µg negatively supercoiled pBR322, DNA gyrase (concentrations were determined in a previously performed enzyme titration and are given in the figures) and different concentrations of inhibitors. Reactions were incubated at 37°C for 60 min prior addition of 3 µL of 2% (*w/v*) SDS and 1.5 µL of 10 mg/mL proteinase K and additional incubation at 37°C for 30 min to trap cleavage complexes. Reactions were subsequently stopped by addition of 30 µL of chloroform/isoamyl alcohol (*v/v*, 24:1) and 30 µL of STEB (40% (*w/v*) sucrose, 100 mM Tris‐HCl pH 8.0, 10 mM EDTA and 0.5 mg/mL bromophenol blue). The aqueous phases of the samples were subsequently loaded on a 1% (*w/v*) agarose gel and the gel electrophoresis was run at 80 V for 2 h. DNA bands were stained with 1 µg/mL ethidium bromide and subsequently quantified using ImageJ software (version 2.3.0). Fitting of the curves and CC_50_ (concentration required to achieve half of maximum DNA cleavage) determination was carried out by sigmoidal curve fitting using GraphPad Prism (version 10.2.3). The same protocol was used to determine the CC_50_ of corramycin for the *Mtb* fusion construct. For the EDTA resealing experiment, cleavage was induced for 30 min at 37°C, followed by addition of 1 µL of a 250 mM EDTA pH 8.0 solution. SDS/proteinase K trapping was performed at the indicated time points as described above. For the negative control and time point 0, cleavage complexes were trapped without prior addition of EDTA.

### 4.18 Determination of the 3D Structure of Corramycin

The 3D structure of corramycin has been determined by restrained MD calculations and energy minimization. MD simulations and interactive modeling were performed using the software package SYBYL. All energy calculations were based on the Tripos force field. For energy minimizations the Powell method was used. The experimental data set used as input for the MD calculations included 107 distance constraints obtained from a ROESY spectrum recorded in methanol‐D3 at 300 K (Figure S19, Tables S10 and S11). Upper and lower distance limits were set to ± 10% of the calculated (experimental) distances, respectively. For methyl groups, 1.0 Å was added to the upper bound as pseudoatom correction. The starting structure was built manually using the biopolymer module of SYBYL. After an initial energy minimization, the ROE derived distance constraints were applied with a force constant of 41.9 kJ mol^−1^ Å^−2^. In the subsequent production run at 300 K, conformers were sampled every 100 ps for a duration of 1000 ps yielding a total of 10 structures for further analysis. The obtained structures were energy‐minimized *in vacuo* with a convergence criterion of 0.21 kJ mol^−1^ Å^−1^.

### 4.19 Cryo‐Electron Microscopy Studies

#### 4.19.1 Nucleic Acid Preparation

A 150 bp DNA duplex was reconstituted using two phosphorylated asymmetric synthetic oligonucleotides obtained from Eurogentec. Oligonucleotides ME‐73b, ME‐77b and their corresponding complementary strands (Table S9) were dissolved in DNase‐free water at 1 mM concentration. The 150 bp double stranded DNA was assembled by mixing at 1:1 molar ratio for each oligonucleotide, annealed by incubating at 95°C for 2 min and then decreasing the temperature by 1°C every 1 min until reaching 20°C.

#### 4.19.2 Nucleoprotein Complex Formation for Cryo‐EM

The purified *Mtb* GyrBA fusion protein was mixed with the 150 bp dsDNA at 1:1 molar ratio with a final concentration of 2 µM. GyrBA, DNA and corramycin mixtures at 40 µM were incubated for 10 min at 37°C. AMPPNP (Sigma) was then added to the ternary complex at 25 µM and further incubated for 30 min at 30°C. The complex was the stored at 4°C until sample freezing on cryo‐EM grids.

#### 4.19.3 Cryo‐EM Sample Preparation

AuF R0.6/1.0 300 mesh grids were glow‐discharged using a Solarus II plasmacleaner (Gatan, Inc), for 10 sec at 5 W prior to the application of 4 µL of the complex. The grids were plunge‐frozen in liquid ethane using a Vitrobot Mk‐IV (Thermo Fisher Scientific) set at 8°C and 100% humidity, blot time 3 s and blot force +20.

#### 4.19.4 Cryo‐EM Data Collection

Cryo‐EM imaging was performed on a Titan Krios3 G4 microscope (FEI) (EMBL, Heidelberg) operated at 300 kV equipped with a Falcon4i camera (Gatan) and a GIF Quantum energy filter (Gatan). The images were recorded in EFTEM nanoprobe mode with Serial EM 4.2.0beta in super‐resolution counting mode with a pixel size of 0.731 Å per pixel, with a defocus range between −2.0 and −0.7 µm. Two datasets with tilt angle of 0° and 30° were collected with a total dose of 50 e^−^/Å^2^ distributed on 40 frames. Statistics for cryo‐EM data collection for the two datasets are listed in Table S7.

#### 4.19.5 Cryo‐EM and Image Processing

All image processing steps were done using CryoSPARC [76]. Movies motion correction was performed using *Patch Motion Correction*. The contrast transfer function (CTF) parameters were estimated using *Patch CTF estimation*. A first round of particle picking done with the *blob picker* tool was used to generate 2D classes that were subsequently used as templates for a second round of template‐based particle picking. After several rounds of 2D classification, the particles from classes displaying high‐resolution features were selected and used to generate reference‐free 3D ab initio models. The particles were further classified in 3D using *Heterogenous Refinement and 3D Classification*. After particles classification, Non‐Uniform (NU) refinement was performed, resulting in a final reconstruction at ∼2.80 Å resolution (Figure S12).

#### 4.19.6 Model Building and Refinement

In an initial step, we fitted the cryo‐EM structure of *Mtb* gyrase cleavage core (PDB: 8S7O), after cleaning the DNA and the BDM71403 molecules, into our map. We manually built the dsDNA using Coot [77] and placed the molecules in the corresponding density. The model was subjected to several cycles of real‐space refinement using Phenix [78, 79].

## Author Contributions

F.F. and S.P. designed the research; J.H., N.R., R.M., J.R., and S.P. supervised the studies; A.H. and U.K. conducted chemical synthesis; J.H., N.R., and J.R. performed myxobacterial extract screening; F.F., M.D.M., and N.R. designed and conducted MIC evaluation; S.A. was in charge of *Mtb* isolates screening; F.F. and M.D.M. performed time‐kill assays; F.F. performed checkerboard assays; G.P. designed and evaluated *Mtb* reporter strains; F.F. and T.R. conducted –omics approaches; F.F. and M.M. performed SEM experiments; F.F. performed DNA displacement assays; F.F. and M.D.M. performed corramycin resistance studies; V.D. and L.S. generated MOX‐resistant mutants and performed analysis. Y.G. and M.D.M. generated recombinant mutants; F.F. performed in vitro biochemical assays; J.B.W., A.C., and A.B. generated the CRISPRi data in experiments supervised by D.S.; M.K. performed NMR and 3D modeling; Y.W. provided purified proteins; Y.W. and F.G. prepared cryo‐EM grids and collected data; Y.W. did the first step of cryo‐EM data processing; A.M. performed cryo‐EM image processing; A.M. and S.P. analyzed cryo‐EM maps, and carried out model building and refinement; F.F. and S.P. prepared figures and wrote the original draft of the manuscript; J.H., N.R., R.M., and J.R. revised the manuscript; all authors completed and edited the paper.

## Funding

This study was supported by the German Center for Infection Research (DZIF; grants TTU02.810, TTU02.814, and TTU 09.827, J.H., U.K., J.R., N.R., R.M.), the German Research Foundation (DFG; grant 545546569, U.K., J.R., R.M.) and by the Gates foundation (grants INV‐001913 and INV‐055900, R.M.; INV‐055896, D.S.). The project was further supported by the IdEx Université Paris Cité (S.P.), by the Agence Nationale de la Recherche (ANR, France) ANR‐24‐CE92‐0080 (S.P.) and by institutional grants from the Centre National de la Recherche Scientifique (CNRS), the Institut Pasteur, and Université Paris Cité.

## Conflicts of Interest

The authors declare no conflicts interest.

## Supporting information




**Supporting File**: advs76832‐sup‐0001‐SuppMat.pdf.

## Data Availability

Mass spectrometry proteomics data have been deposited to the ProteomeXchange Consortium via the PRIDE partner repository with the dataset identifier PXD060283. WGS data are available at the National Center for Biotechnology Information (NCBI) through the BioProject accession numbers PRJNA1263475 and PRJNA1354811 (corramycin), as well as PRJNA1381569 (FQs). RNASeq data were deposited in the NCBI Gene Expression Omnibus (GEO) and are accessible through the GEO series accession number GSE310369. The corramycin‐bound *Mtb* gyrase coordinates have been submitted to the Protein Data Bank with the PDB ID 9QUT. The corresponding EM map has been submitted to Electron Microscopy Data Bank with ID EMD‐53381. Other data are available from corresponding authors upon reasonable request.
