# Supplementary material for: The Natural Product Corramycin Acts as a DNA Gyrase Poison and Overcomes Fluoroquinolone Resistance in Mycobacterium tuberculosis
Source: Adv Sci (Weinh). 2026 Aug 10;13(63):e76832. doi: 10.1002/advs.76832 (PMC13455829; doi:10.1002/advs.76832)
Supplement: Supplementary file 1 [file ADVS-13-e76832-s001.pdf]

Supplementary Information

The Natural Product Corramycin Acts as a DNA Gyrase Poison and Overcomes Fluoroquinolone Resistance in *Mycobacterium tuberculosis*

Supplementary Figures

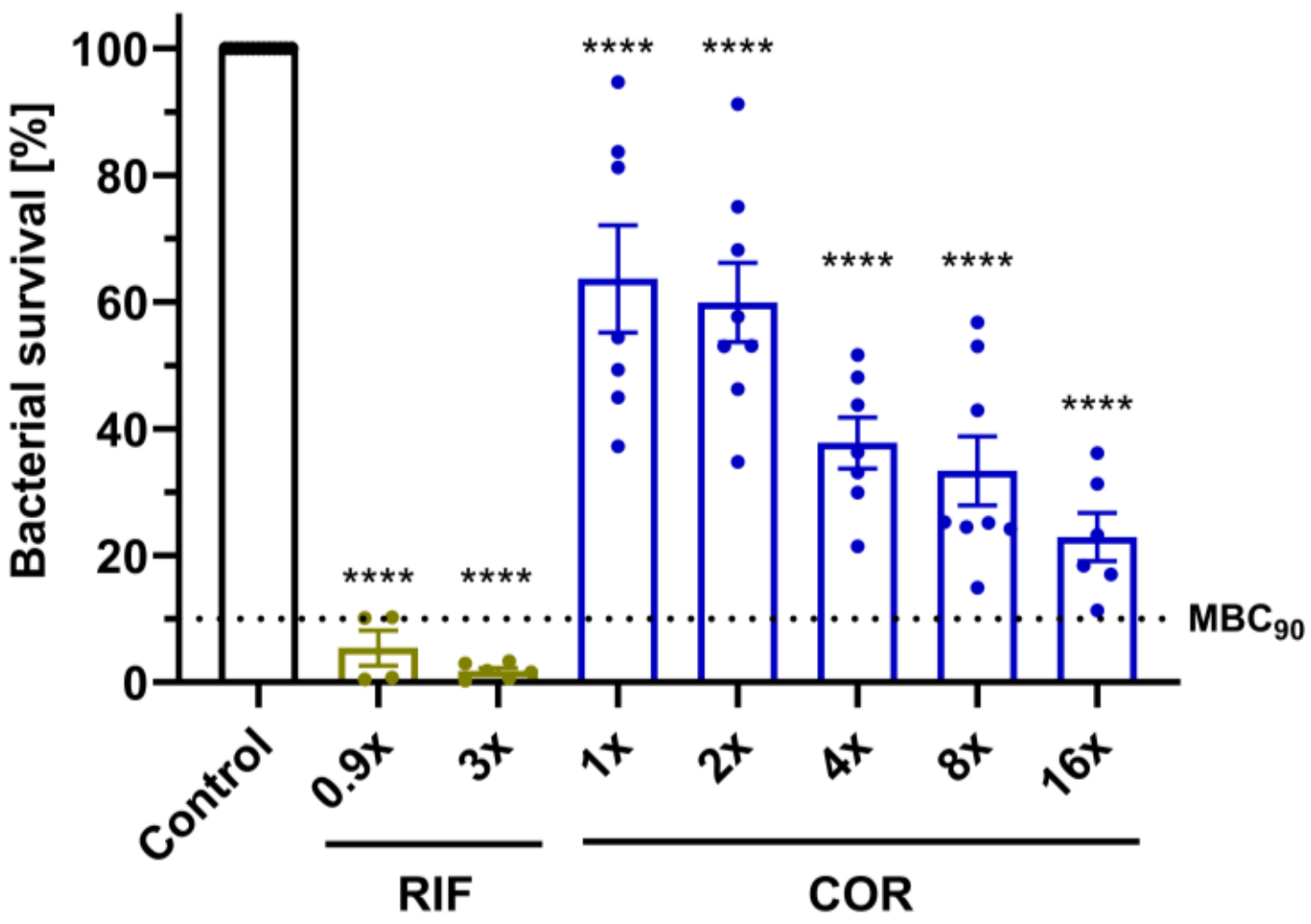

Figure S1. Activity of corramycin against intracellular *M. tuberculosis* H37Rv in human primary macrophages. Macrophages were infected with *M. tuberculosis* cells at a multiplicity of infection (MOI) of 0.5 and infected cells were treated with corramycin or rifampicin at the indicated concentrations (MIC multiples). The viability of intracellular *M. tuberculosis* was determined 7 days post infection by cell counting and data was normalized to an untreated control. Data represent mean values ± SEM from at least six independent biological replicates performed with human primary macrophages derived from different donors. The horizontal dotted line indicates the minimum bactericidal concentration required to achieve 90% killing relative to the untreated control (MBC_{90}). Statistical analysis was performed by repeated-measures one-way ANOVA with Dunnett's multiple comparison test. $p < 0.0001$: ****. COR, corramycin; RIF, rifampicin.

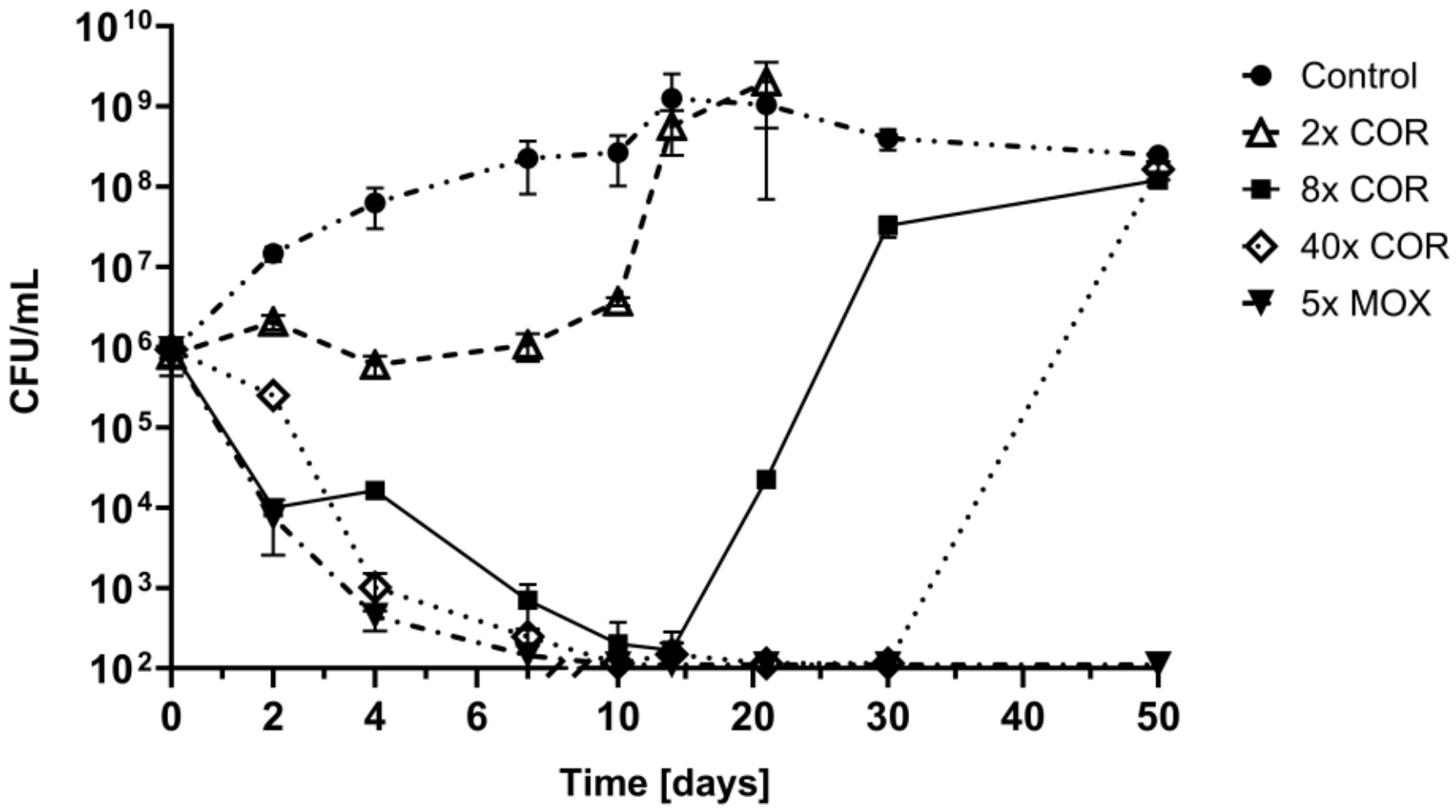

Figure S2. Time- and concentration-dependent killing of *Mtb* Erdman. The control represents *Mtb* exposed to DMSO. Limit of detection is 10^2 CFU/mL. Data represent mean ± SD ($n = 2$). COR, corramycin; MOX, moxifloxacin.

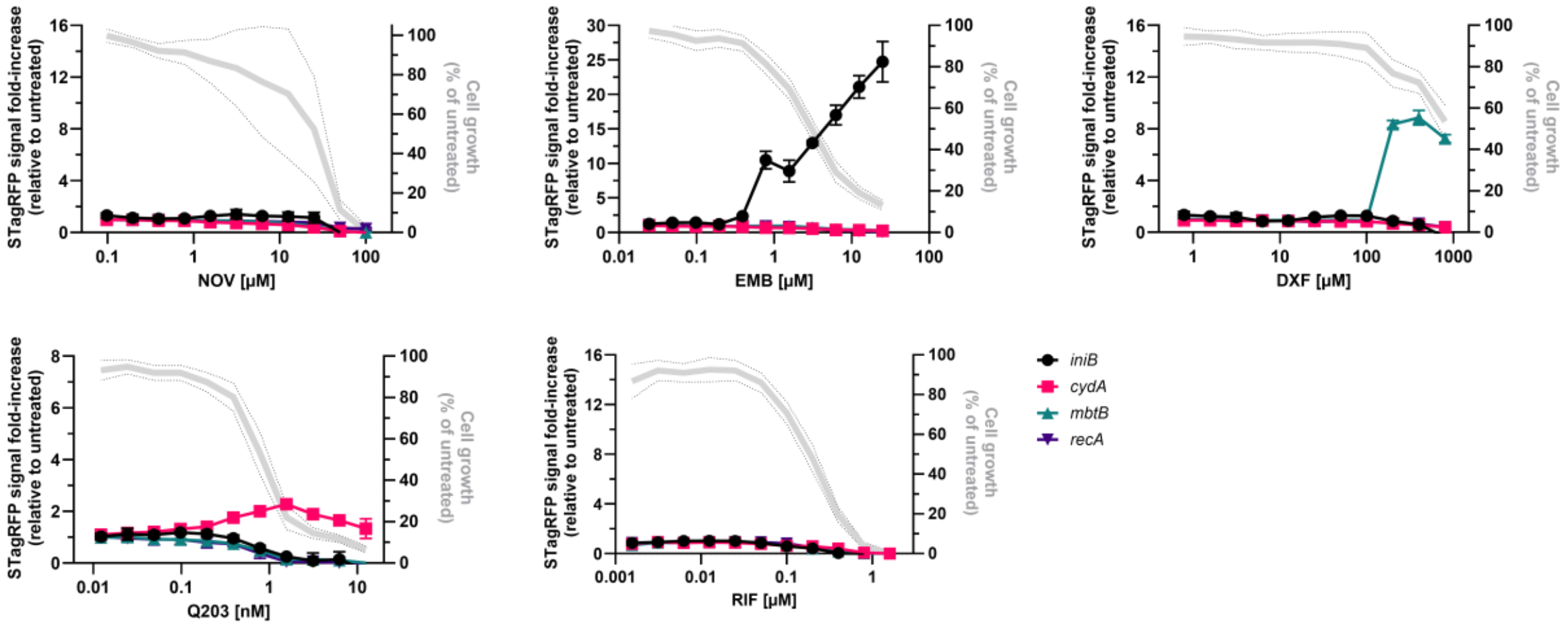

Figure S3. Validation of the fluorescent reporter assay with reference compounds known to cause iron deprivation (*mbtB*), DNA damage (*recA*), disruption of cell wall biosynthesis (*iniB*) or of mycobacterial respiration (*cydA*). Data represent mean values ± SD of two biological replicates (*n* = 2). DXF, deferoxamine; EMB, ethambutol; NOV, novobiocin; RIF, rifampicin.

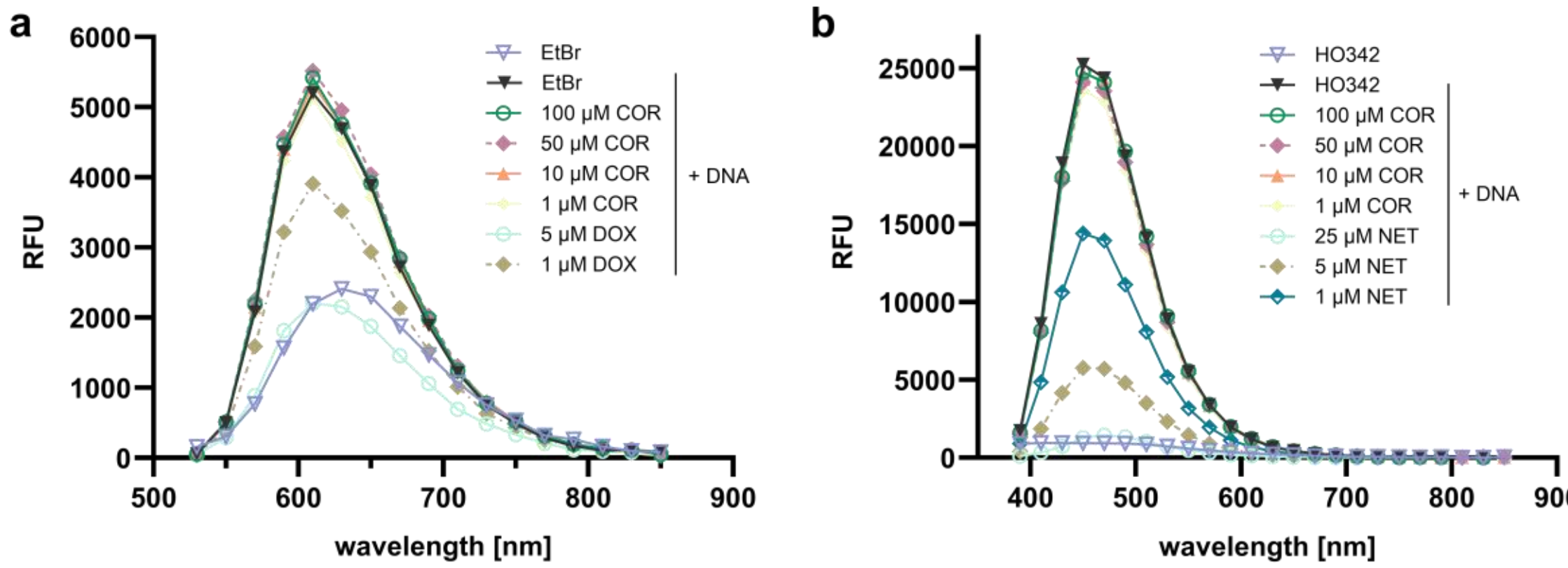

Figure S4. DNA displacement assays. No displacement of ethidium bromide (**a**) or Hoechst33342 (**b**) was observed, thus corramycin does not intercalate between DNA bases or binds to the minor groove of the DNA. Doxorubicin and netropsin were used as positive controls. Data represent the average relative fluorescence units (RFU) of ethidium bromide/Hoechst33342 for one representative biological replicate (*n* = 2). COR, corramycin; DOX, doxorubicin; EtBr, ethidium bromide; HO342, Hoechst33342; NET, netropsin.

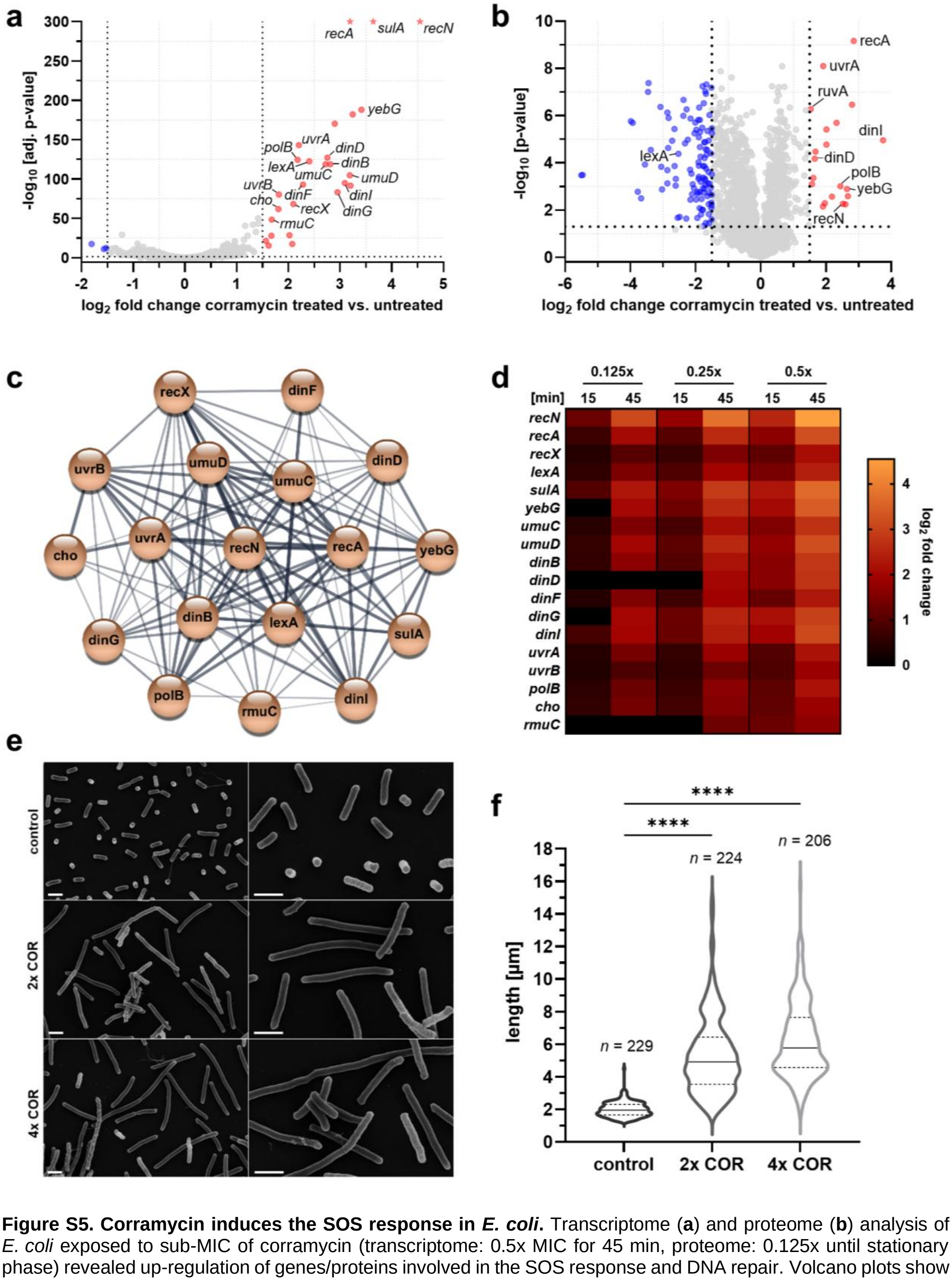

Figure S5. Corramycin induces the SOS response in *E. coli*. Transcriptome (**a**) and proteome (**b**) analysis of *E. coli* exposed to sub-MIC of corramycin (transcriptome: 0.5x MIC for 45 min, proteome: 0.125x until stationary phase) revealed up-regulation of genes/proteins involved in the SOS response and DNA repair. Volcano plots show RNAs/proteins with significant changes in expression ($\log_2$ fold change ≥ 1.5, p-value < 0.05). Significance was determined by the Wald test (transcriptomics, *n* = 3) and by two-tailed student's T-test (proteomics, *n* = 4). Each point represents the average value of one transcript/protein. Asterisks represent imputed values for which the p-value was recorded as 0. **c,** Network cluster of SOS response and DNA repair genes identified in the full STRING network of up-regulated genes. **d,** Heat map of differentially expressed genes belonging to the local STRING network shown in **c**. **e,** Scanning electron microscopy (SEM) of *E. coli* exposed to 2x and 4x MIC of corramycin (T = 2 h). Scale bars are 2 µm. **f,** Violin plot showing cell elongation in the presence of corramycin. Significance was determined by Kruskal-Wallis test. p < 0.0001: ****. COR, corramycin.

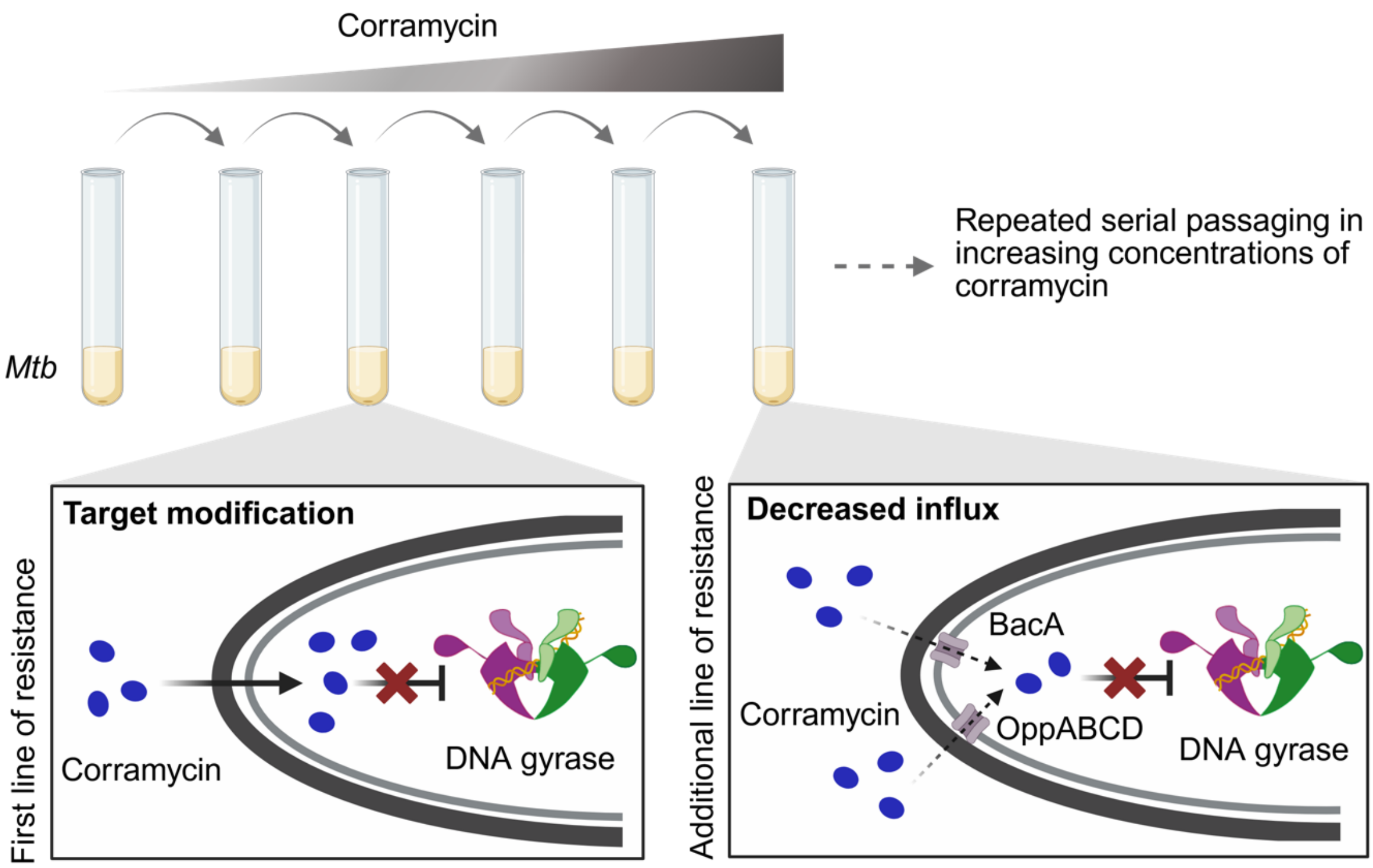

Figure S6. Evolutionary selection of corramycin-resistant *M. tuberculosis* by serial passaging. Whole genome sequencing revealed gyrase mutations as first line of defense and importer mutations as additional line of defense. Created in BioRender. Fries, F. (2026) https://BioRender.com/bc1xko5.

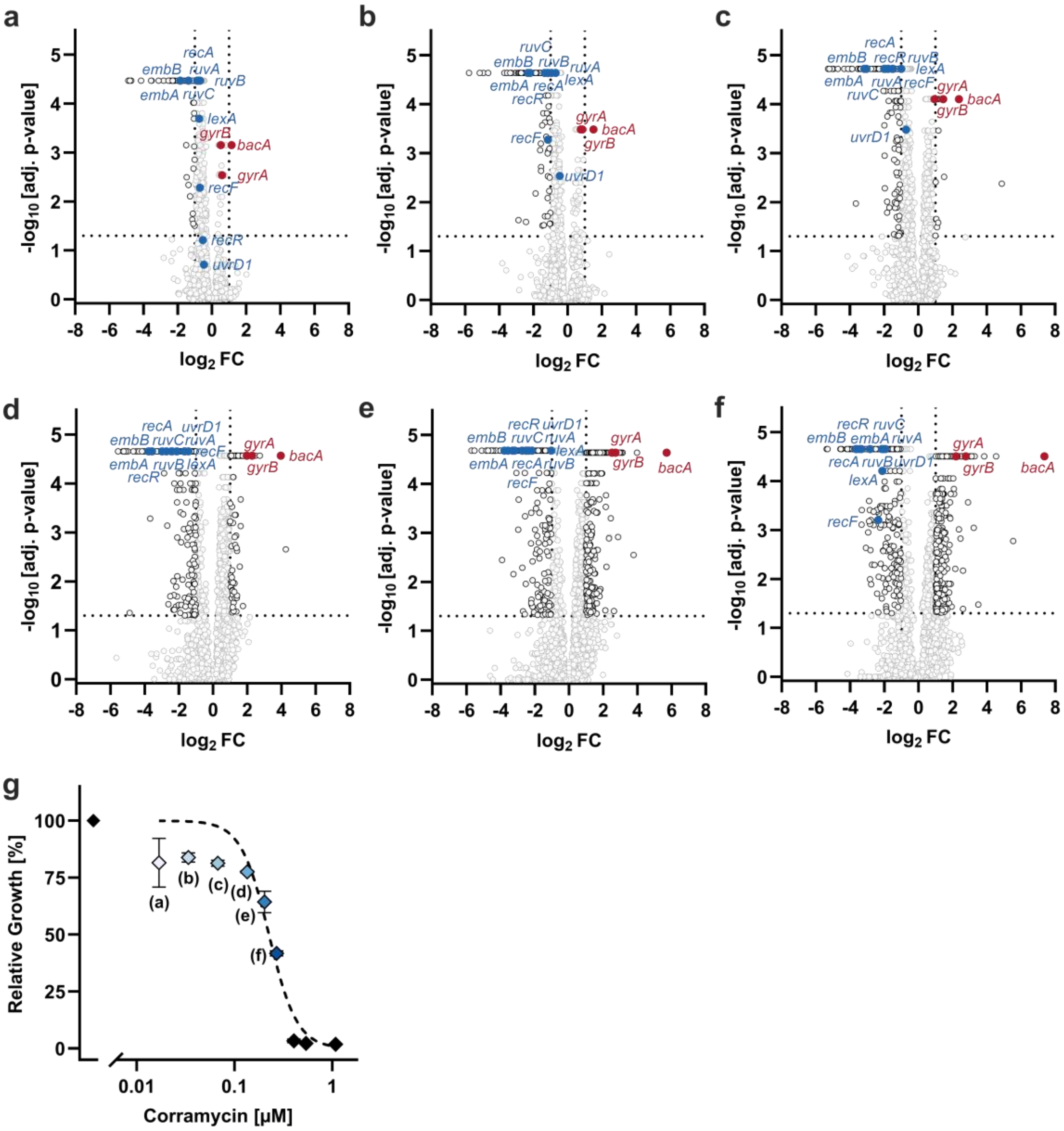

Figure S7. Screening of a genome-wide CRISPRi library to identify genes that influence corramycin efficacy in *M. tuberculosis*. Volcano plots showing log_2 fold change (FC) values and p-values (n = 3) for each gene for the indicated drug concentrations (**a,** 0.03x, **b,** 0.06x**, c,** 0.125x, **d,** 0.25x, **e,** 0.375x and **f,** 0.5x MIC as indicated in the growth inhibition curve depicted in **g**).

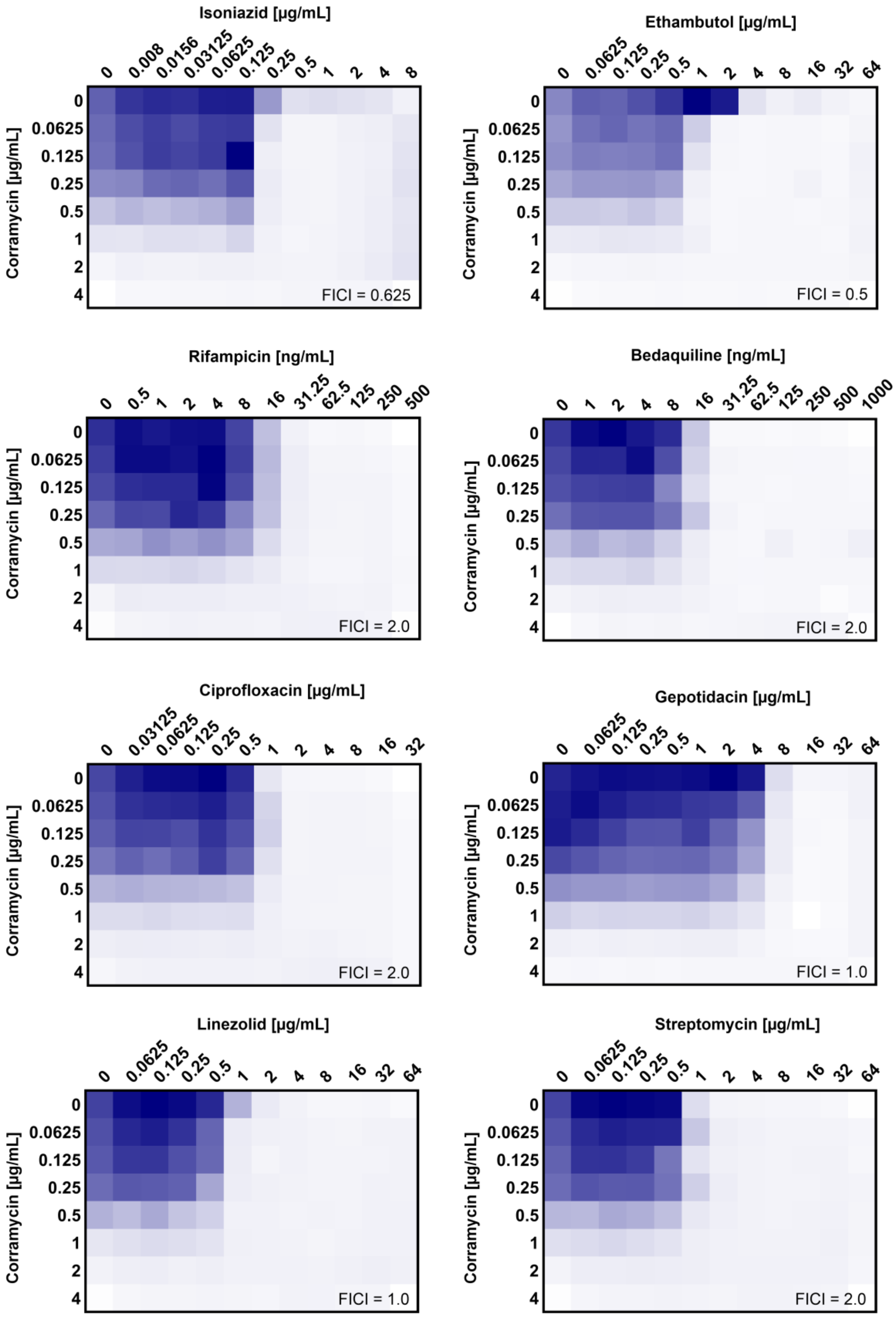

Figure S8. Heat map depiction of checkerboard assays in *M. tuberculosis* H37Ra. Data represent the relative fluorescence of resazurin in each well for one representative biological replicate ($n = 2$). Dark blue regions represent higher cell density. Synergy is defined as FIC index ≤ 0.5. Corramycin shows a weak synergistic effect with the mycobacterial cell wall inhibitor ethambutol, suggesting that a weakened cell wall might facilitate the uptake of the natural product, leading to increased intracellular concentrations and thus, enhanced potency.

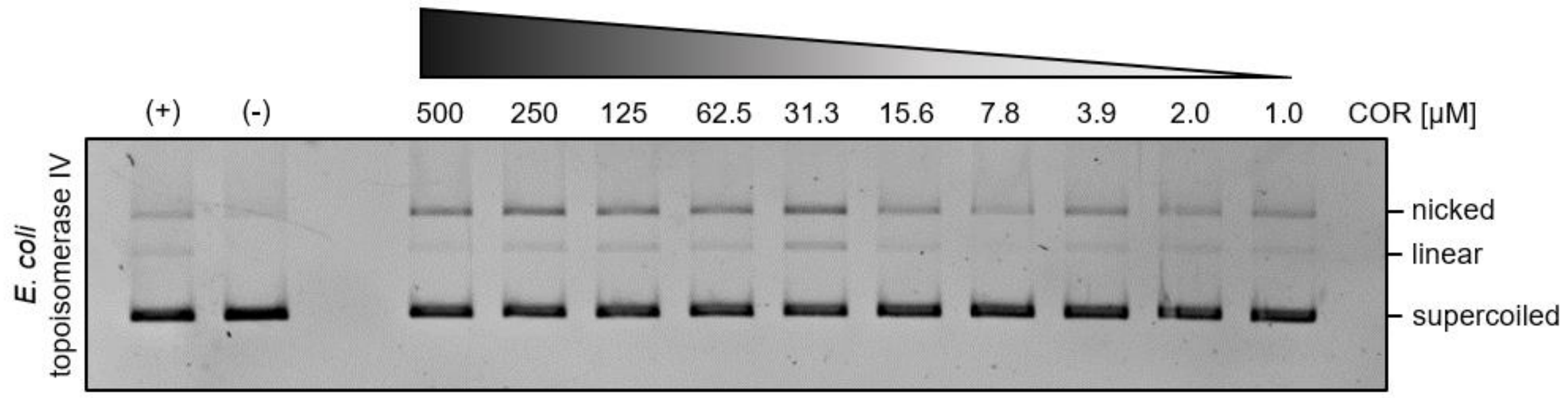

Figure S9. DNA cleavage assay for *E. coli* topoisomerase IV. Representative agarose gel with indicated positions for nicked, linear and supercoiled plasmid. (+) represents a positive control containing topo IV (53 nM) without inhibitor, while (-) represents a negative control without topo IV and without inhibitor. COR, corramycin.

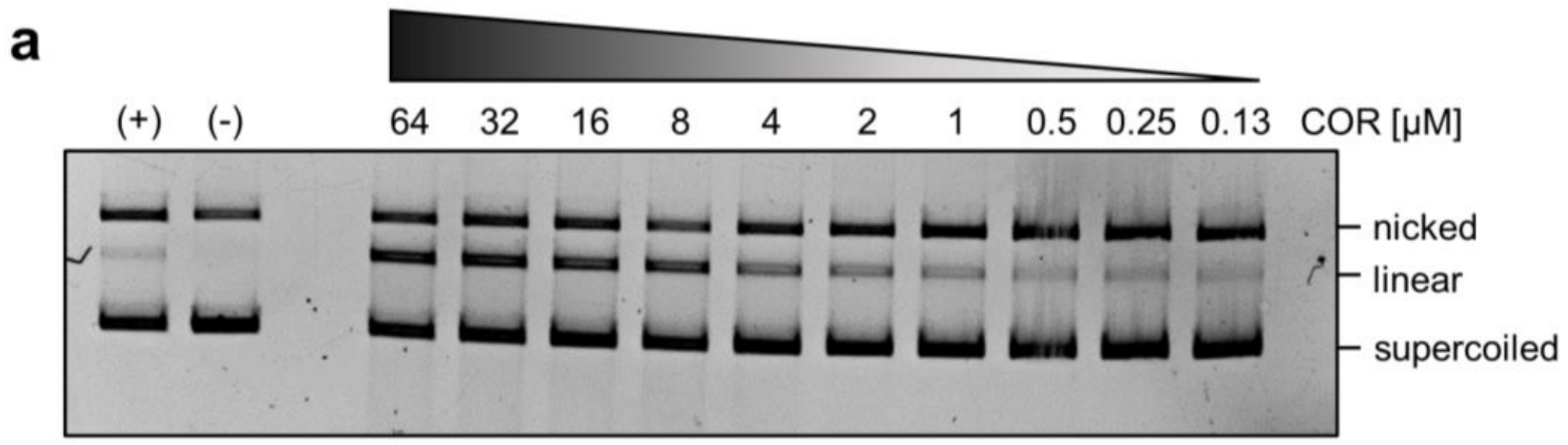

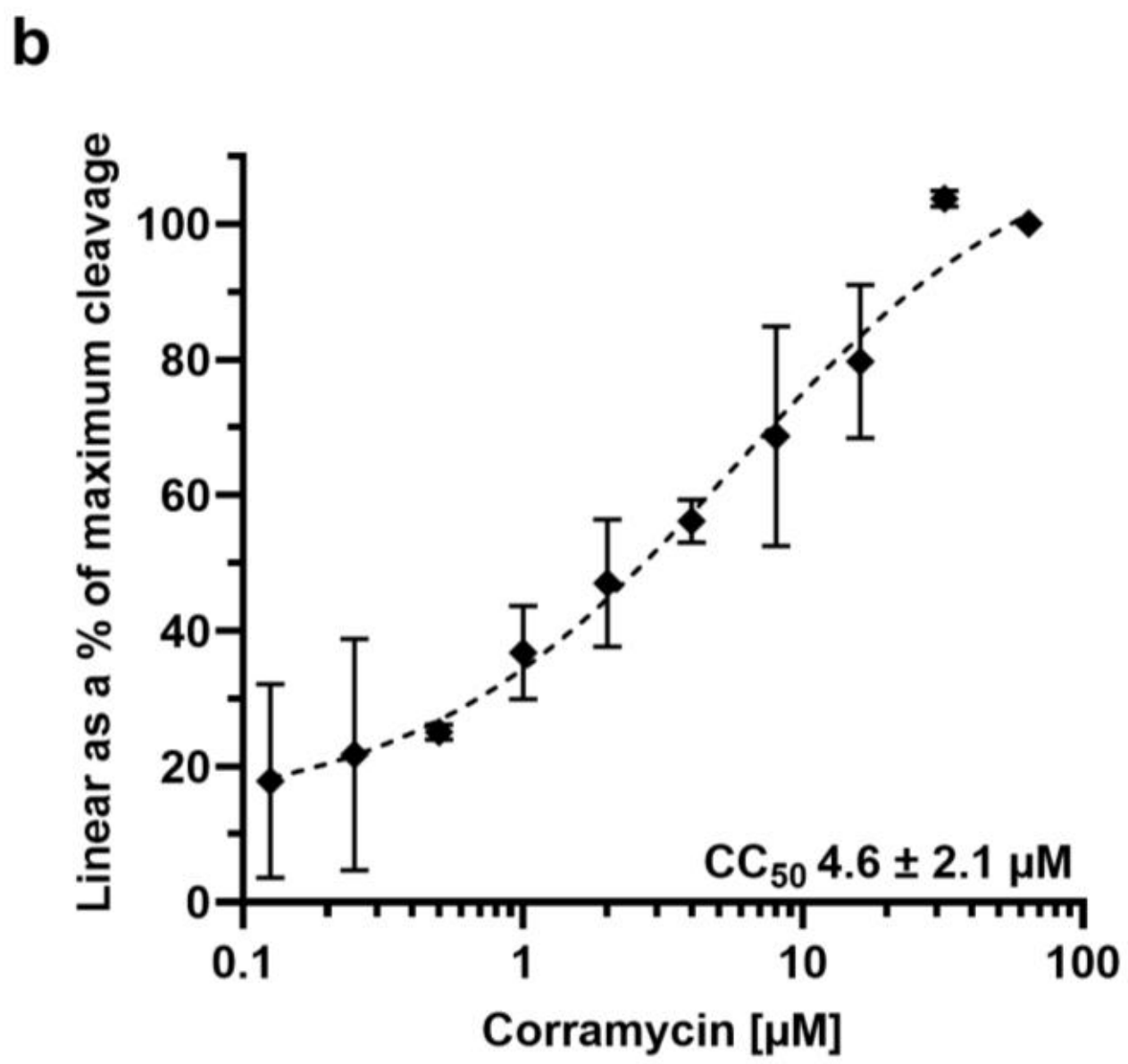

Figure S10. Cleavage assay for *S. aureus* DNA gyrase. a, Representative agarose gel with indicated positions for nicked, linear and supercoiled plasmid. (+) represents a positive control containing *S. aureus* gyrase (53 nM) without inhibitor, while (-) represents a negative control without gyrase and without inhibitor. **b,** Plot used for CC_{50} determination. Values are plotted as mean values ± SD (n = 3). Line represents sigmoidal fit to the data. COR, corramycin.

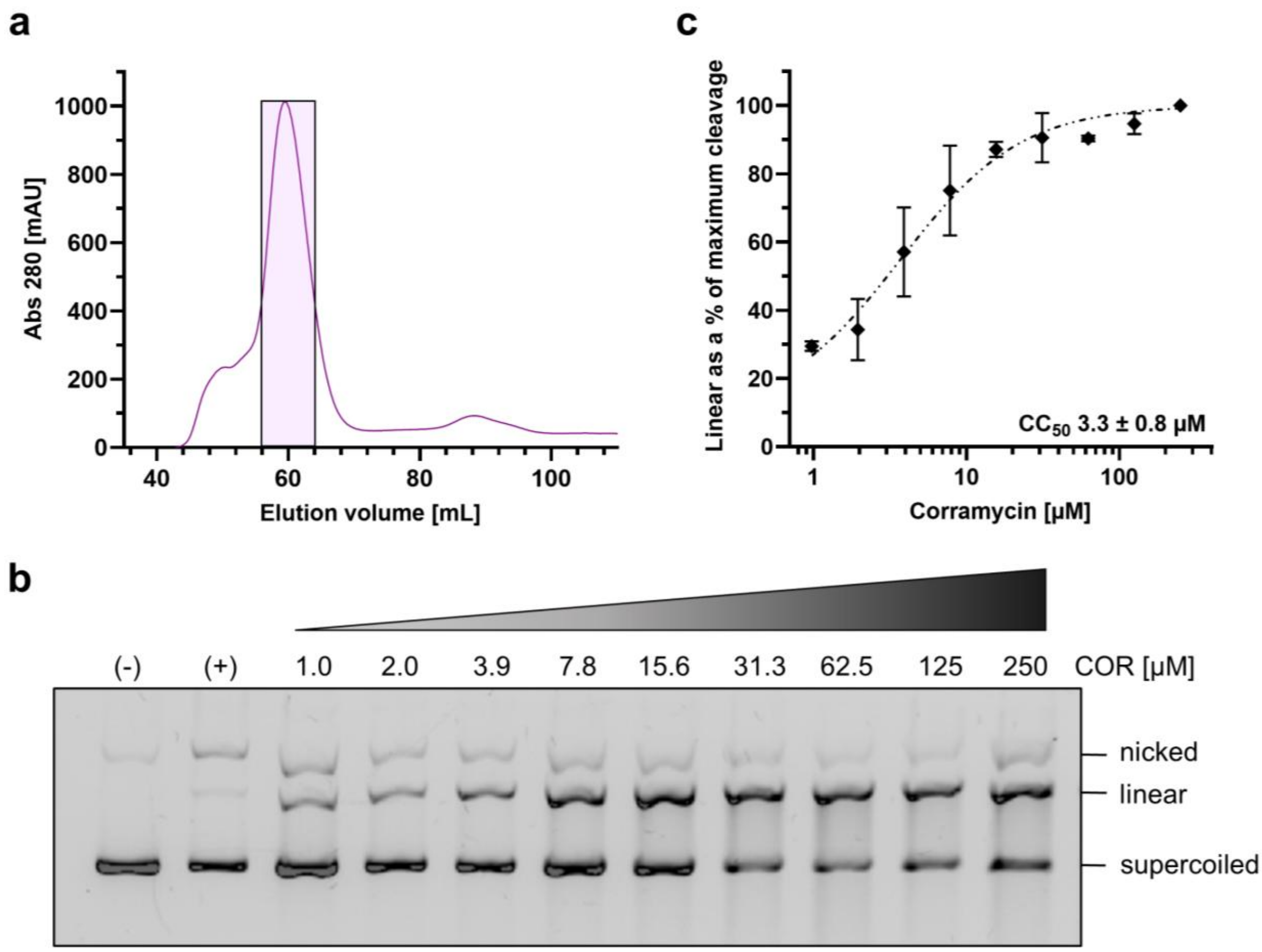

Figure S11. Biochemical characterization of the *Mtb* GyrBA fusion construct. a, Size-exclusion chromatogram (Superdex S200 16/60) corresponding to the final purification step of the *Mtb* GyrBA fusion protein. The box highlights the collected fractions. **b,** Representative agarose gel of a cleavage assay with 47 nM *Mtb* GyrBA. Positions for nicked, linear and supercoiled plasmid are indicated. (+) represents a positive control containing gyrase without inhibitor, while (-) represents a negative control without gyrase and without inhibitor. **c,** Plot used for CC_{50} determination. Values are plotted as mean values ± SD (n = 2). Line represents sigmoidal fit to the data. COR, corramycin.

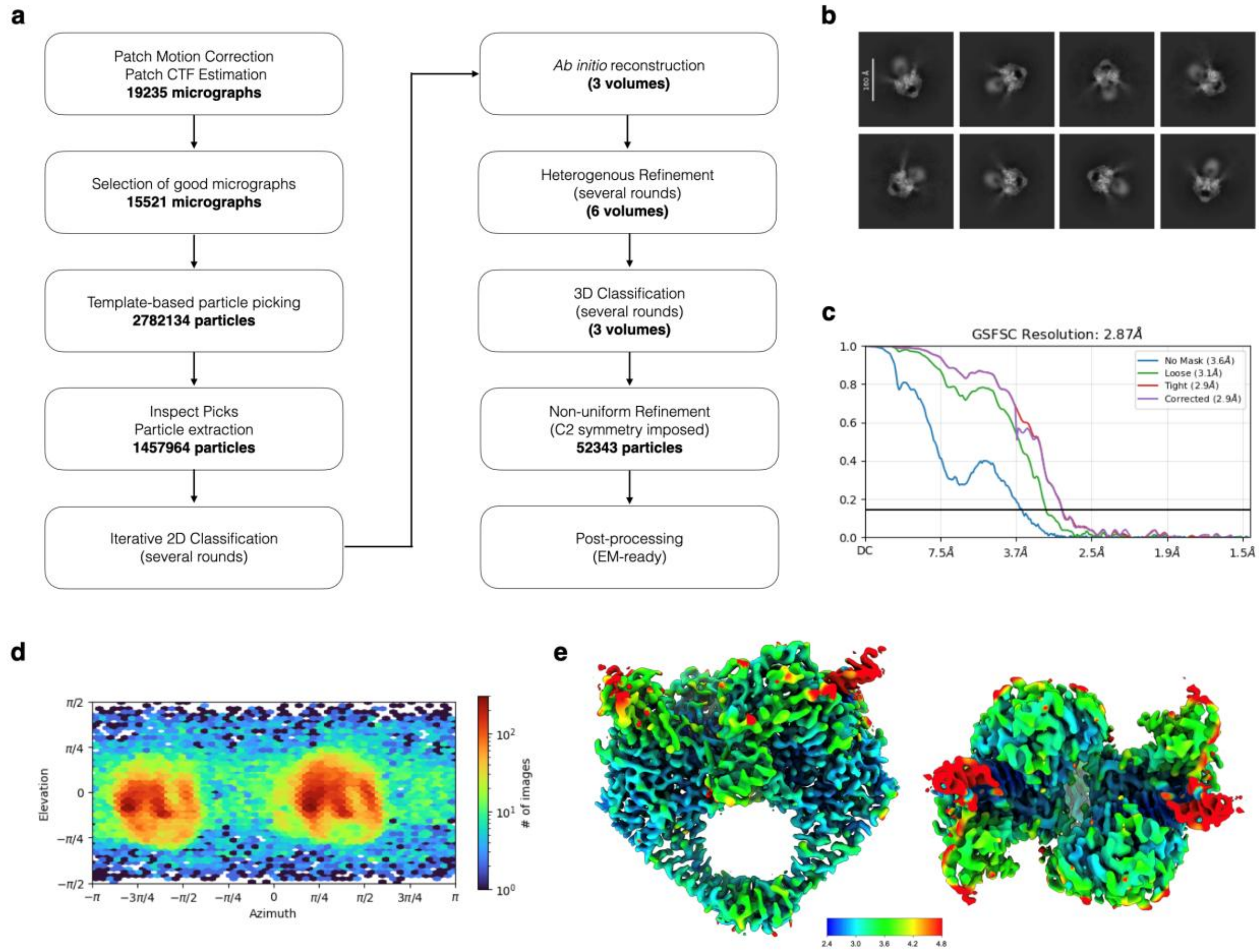

Figure S12. Cryo-EM data processing. a, Schematic diagram illustrating the cryo-EM data processing procedure. **b,** Representative 2D classes. **c,** Fourier shell correlation (FSC) curve. **d,** Euler angle distribution. **e,** Local resolution maps illustrate resolution distribution from 2.4 Å next to the DNA and corramycin molecules, to > 4 Å towards the end of the DNA.

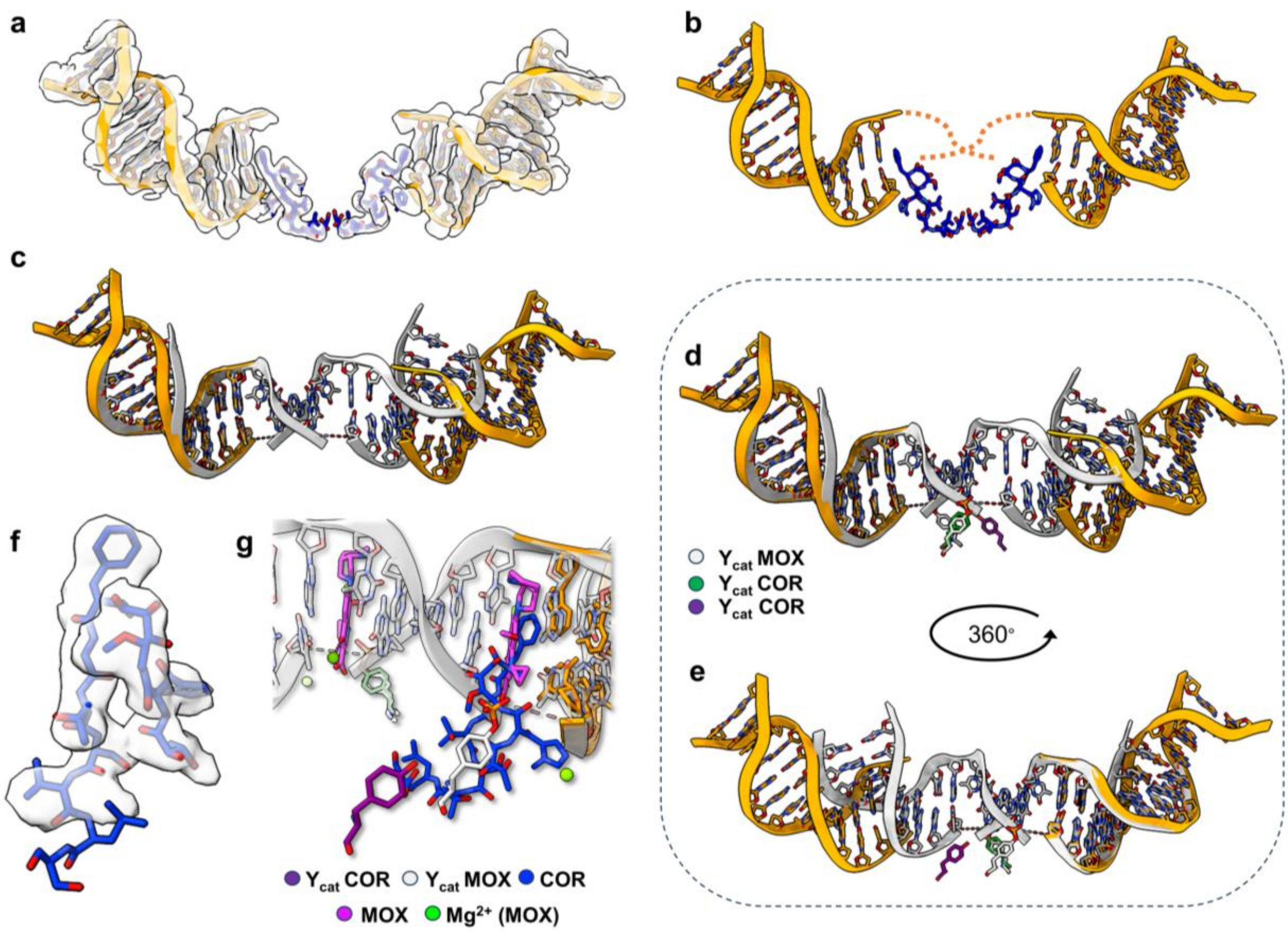

Figure S13. State of the dsDNA. a, High resolution cryo-EM map for DNA (orange) and corramycin (blue). **b,** DNA state and localization of the two COR molecules in the COR-complex. The lacking bases are indicated with orange dots. **c,** Comparison of the DNA states in the COR-complex (orange, current study) and the MOX-complex (light gray, PDB: 5BS8).[1] **d,e** Positions of the corresponding catalytic tyrosines (Y_{cat}) in the COR- and MOX-complexes. **f,** High resolution cryo-EM map for corramycin (blue). **g,** Comparison of COR (blue) and MOX-binding pockets (magenta) inside the DNA. COR, corramycin; MOX, moxifloxacin.

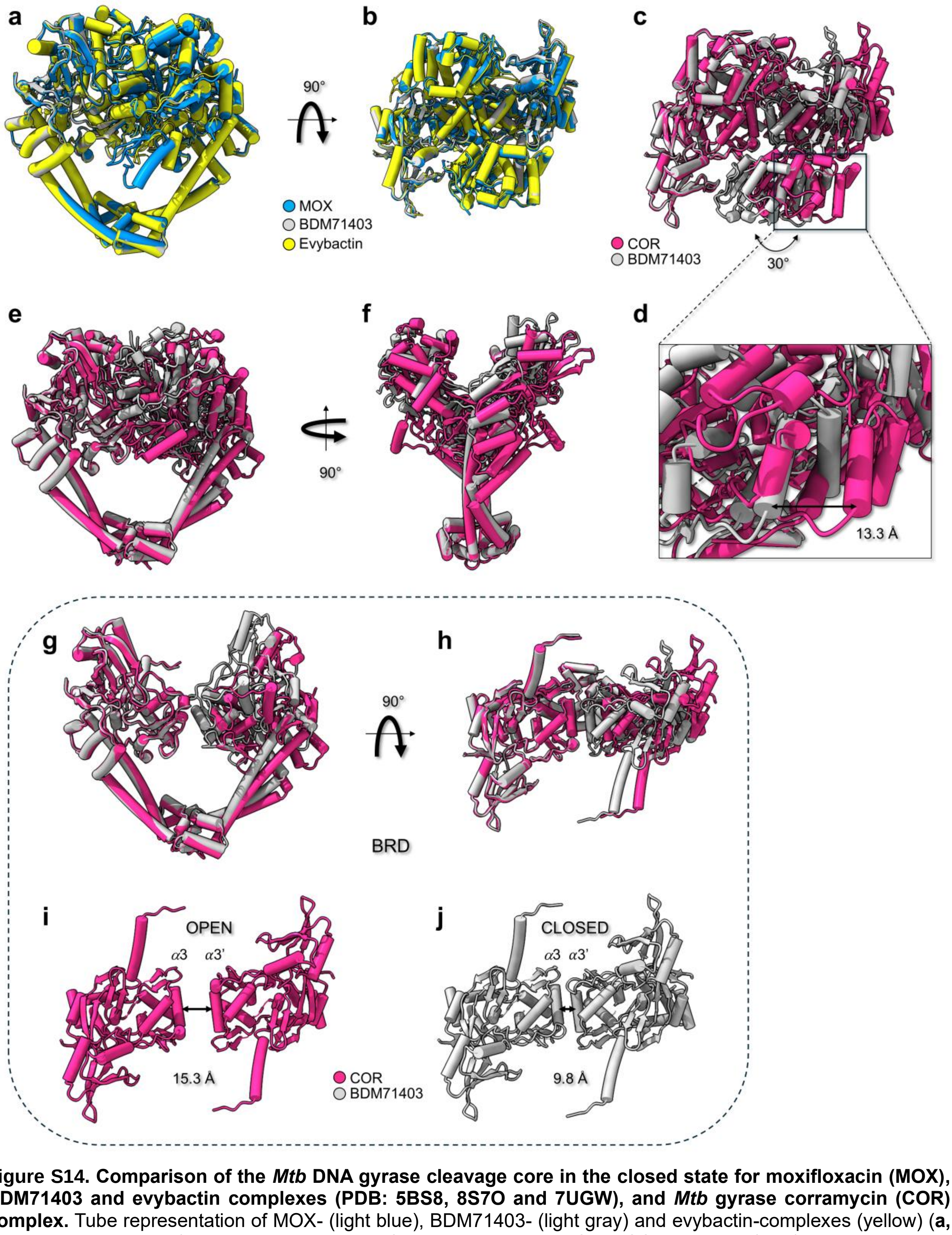

Figure S14. Comparison of the *Mtb* DNA gyrase cleavage core in the closed state for moxifloxacin (MOX), BDM71403 and evybactin complexes (PDB: 5BS8, 8S7O and 7UGW), and *Mtb* gyrase corramycin (COR) complex. Tube representation of MOX- (light blue), BDM71403- (light gray) and evybactin-complexes (yellow) (**a,** front view, **b,** top view). Tube representation of the cleavage core of the COR-complex (pink) overlaid with the cleavage core of the BDM71403-complex (light gray) (**c,** top view, **d,** zoom on the rotation of the TOPRIM domains (GryB), **e,** front view, **f,** side view). Overall opening of the enzyme consists of movements of TOPRIM (downward rotation) and opening doors movement of the breakage-reunion domain (BRD, GyrA, see panels **g-j**).

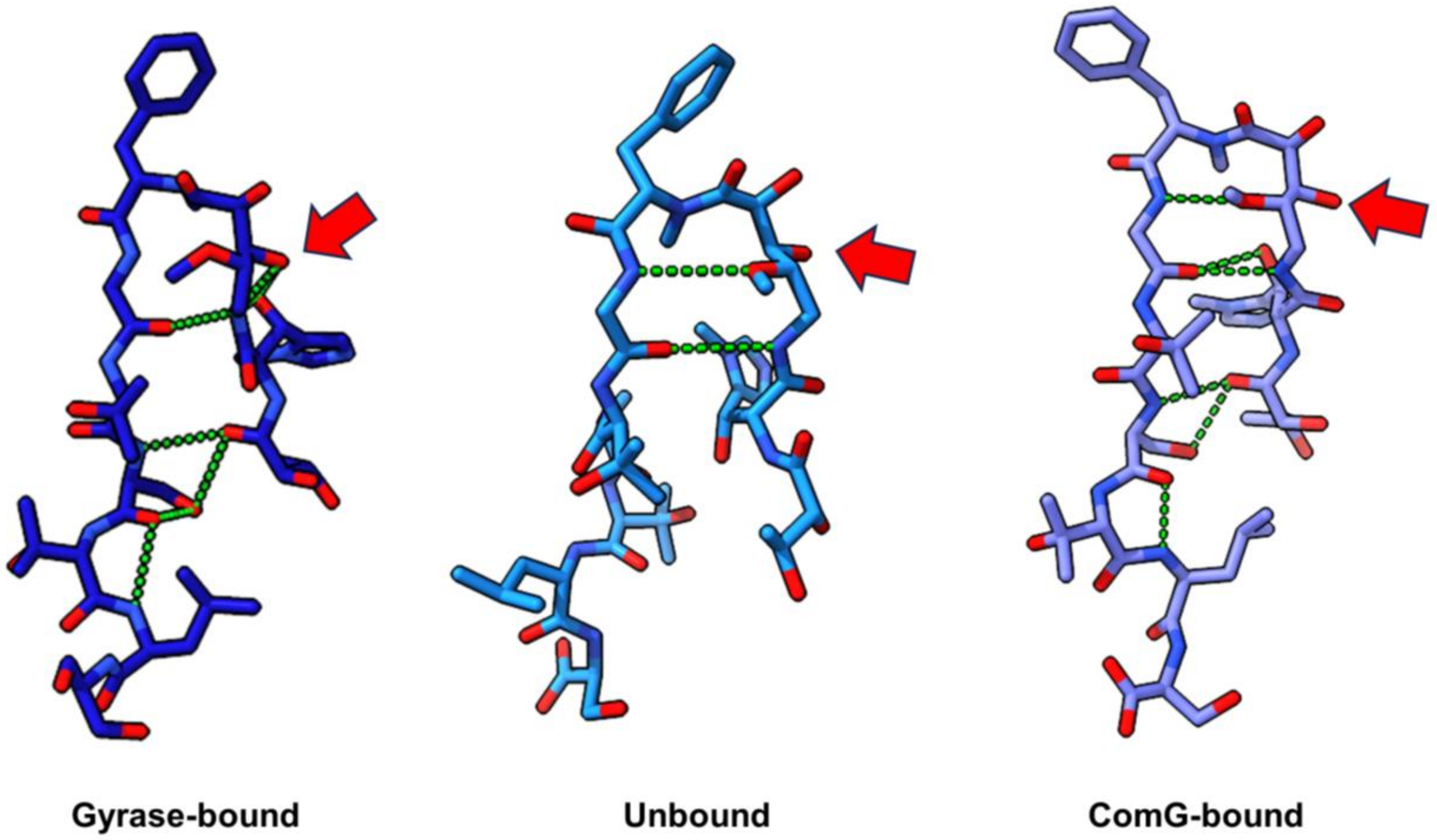

Figure S15. Intramolecular stabilization of corramycin. The drug adopts a V-shaped orientation under all conditions, which is stabilized by intramolecular hydrogen bonds (shown as green dashes). The red arrow indicates the site of phosphorylation when corramycin is in its inactive form (as part of the self-resistance of the producer[2]). The gyrase-bound form is from the current study as the unbound form, the ComG-bound form is extracted from the PDB 8AGY.

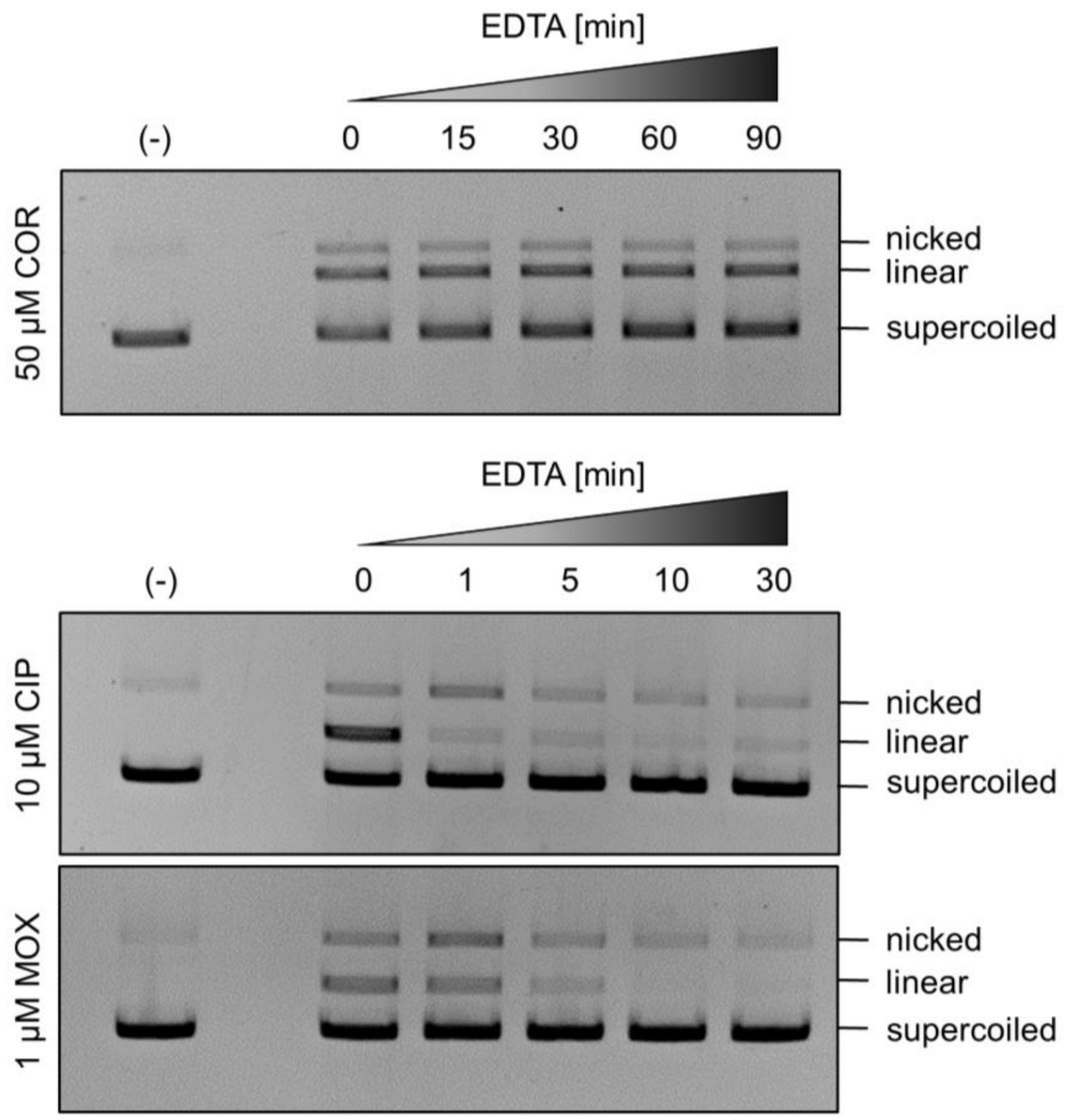

Figure S16. Stability of FQ and corramycin cleavage complexes in the presence of EDTA. Agarose gels showing *Mtb* gyrase DNA cleavage reactions using 47 nM *Mtb* gyrase and 50 µM corramycin, 10 µM ciprofloxacin, or 1 µM moxifloxacin, followed by addition of EDTA for the indicated times. (-) represents a negative control without gyrase and without inhibitor. CIP, ciprofloxacin; COR, corramycin; MOX, moxifloxacin.

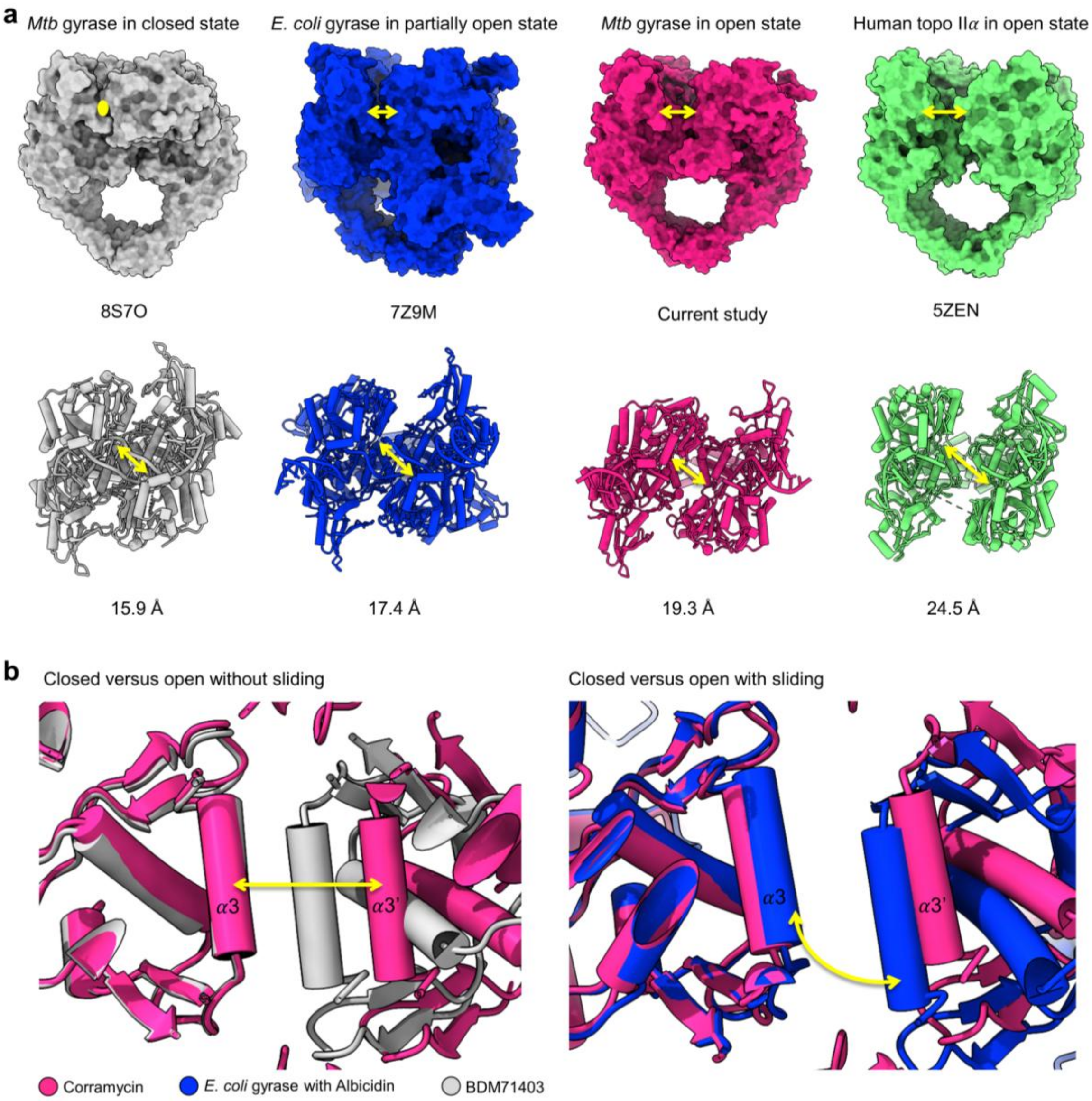

Figure S17. Structural changes at the level of the cleavage core. a, Surface representation for the front view and cartoon representation for the top view of the cleavage cores; of the closed conformation of *Mtb* gyrase (light gray), the partially open conformation of *E. coli* gyrase (blue), the open conformation of *Mtb* gyrase (pink) and the open conformation of the human topo IIα (green). The corramycin-bound structure represents a previously uncharacterized catalytic intermediate that can be placed between the closed state observed in the BDM71403 complex (PDB: 8S7O), the partially open state of the *E. coli* gyrase-albicidin complex (PDB: 7Z9M) and the fully open conformation of human topoisomerase IIα (PDB: 5ZEN).[3,4] **b,** Zoom on the α3/α3' doors of the BRD in cartoon representation. The colors are the same as in panel **a**. The key distinction among these three "open states" is the absence of quaternary sliding of the two halves of the cleavage core in the corramycin-bound complex.

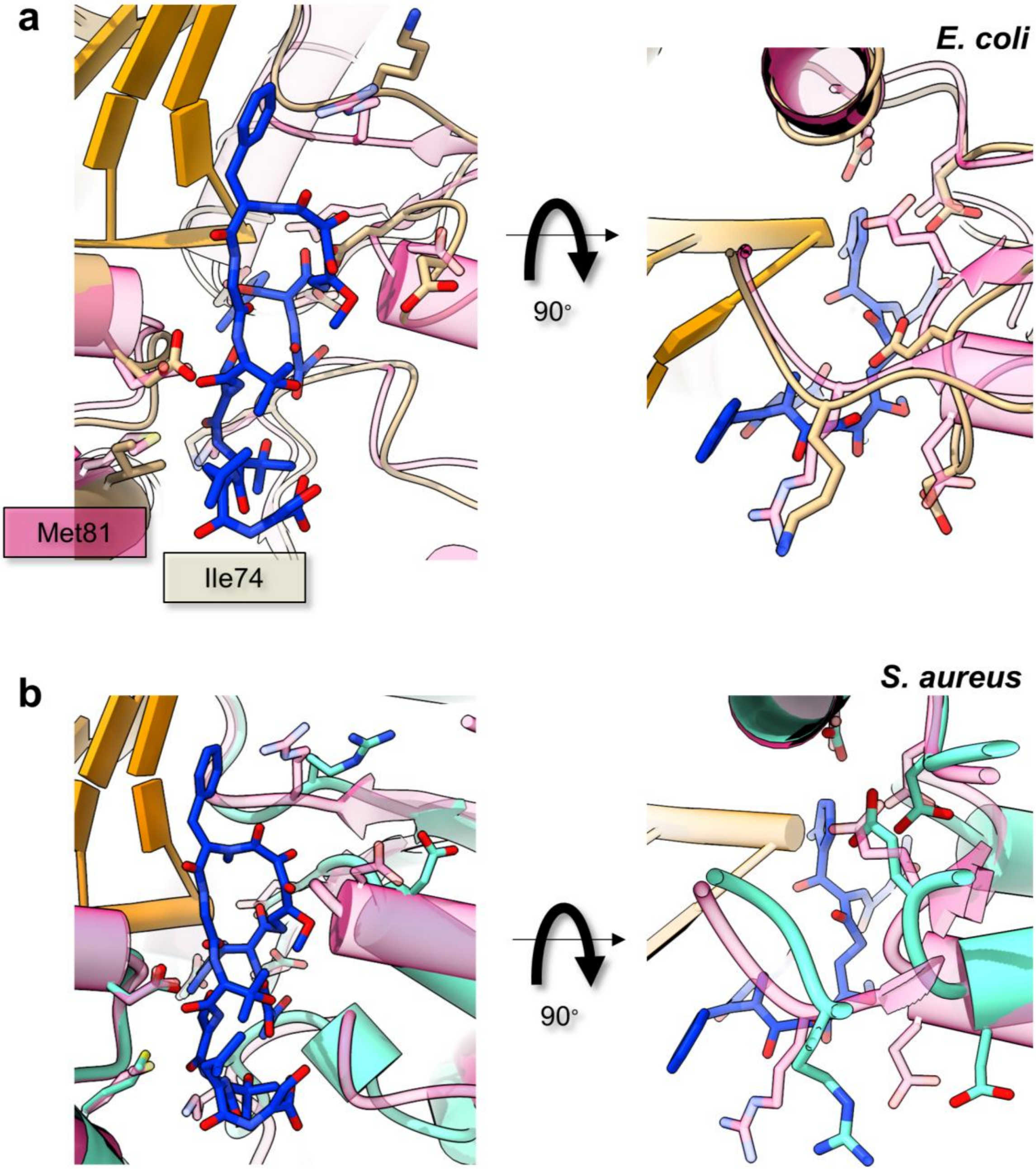

Figure S18. Comparison of corramycin binding pockets between Gram-negative and Gram-positive species. a, Comparison of corramycin binding pocket in *Mtb* (this study, gyrase in pink, DNA in orange) and a generated model of *E. coli* gyrase (PDB: 6RKW, in beige). Notable residues Met81 and Ile74 are indicated. No other significant differences were found in the neighbouring residues. **b,** Comparison of corramycin binding pocket in *Mtb* (this study, gyrase in pink, DNA in orange) and a generated model of *S. aureus* gyrase (PDB: 2XCS, in light blue). No significant differences were observed in the corramycin binding pocket residues. For all comparisons, models were superimposed using ChimeraX matchmaker function.

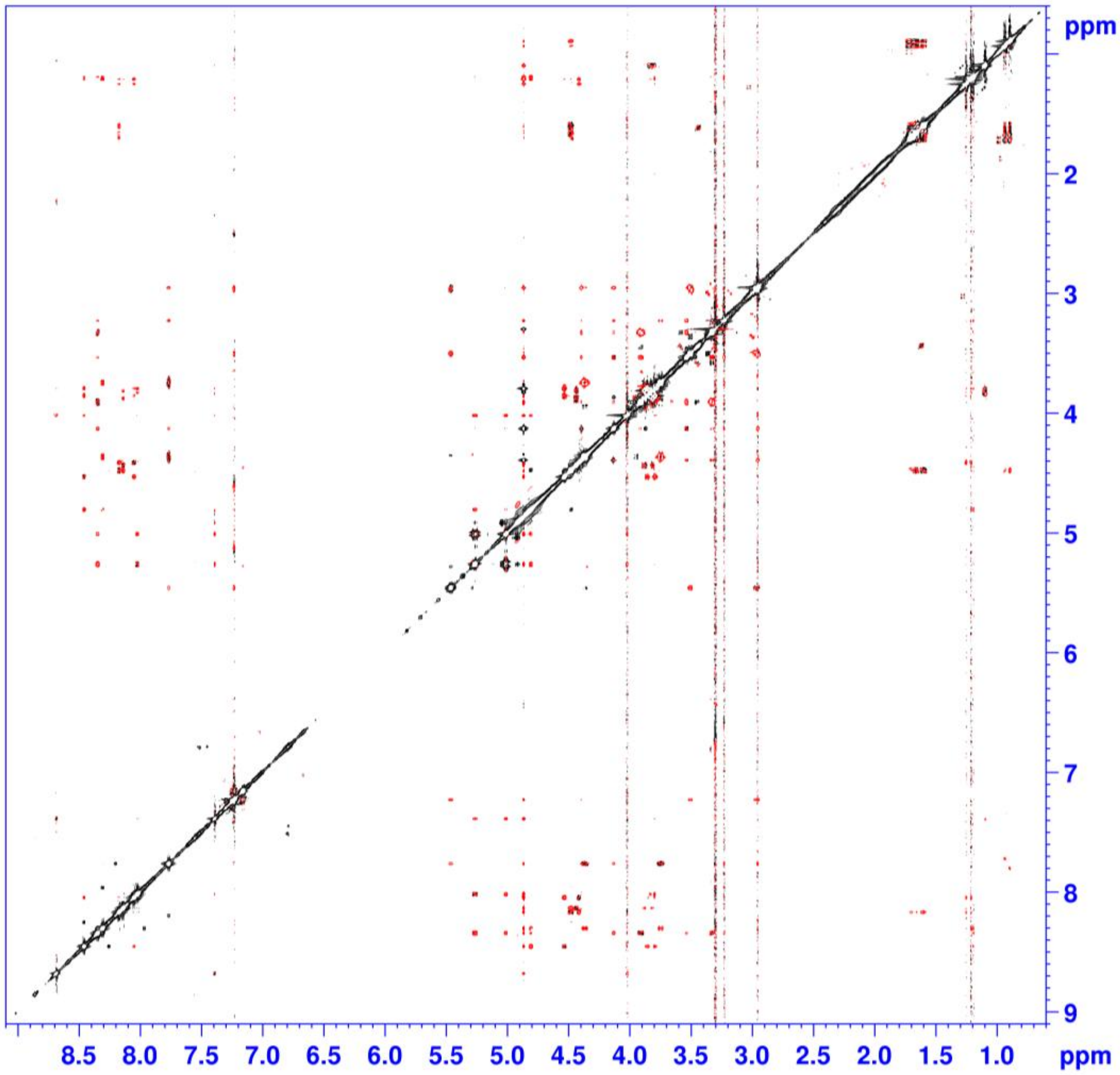

Figure S19. ROESY spectrum of corramycin in methanol-D3 at 300 K. Presaturation has been used for solvent suppression. The spectrum has been acquired with 4096 data points in F2 and 1536 increments in F1 using a mixing time of 150 ms. The spectrum was recorded on a Bruker AVANCE III equipped with a TXI cryoprobe operating at a proton frequency of 700.20 MHz. The ROE correlations have been integrated and transferred into distance constraints used for the MD simulations (Table S11).

Supplementary Tables

Table S1. Spectrum of corramycin. Minimum inhibitory concentrations (MICs) were determined using the broth microdilution method in accordance with EUCAST guidelines. Half-maximal inhibitory concentrations (IC_{50}) were determined using an MTT assay. Values represent a minimum of two independent repeats. PMBN, polymyxin B nonapeptide.

**Organism**	**MIC [µM]**
E. coli BW25223	8
E. coli ATCC25922	4
E. coli DSM19206	4
E. coli DSM26863	2
E. coli ∆*sbmA*	> 64
E. coli WO153	0.5
E. coli K12 ∆*tolC*	0.5
E. coli K12 ∆*tolC* + 1.5 µg/mL PMBN	0.0625
E. coli JW0451-2 ∆*acrB*	2
S. enterica Typhimurium DSM5569	4
S. enterica Typhimurium SB300	8
K. pneumoniae DSM681	8
K. pneumoniae DSM30104	64
A. baumannii ATCC17978	> 64
P. aeruginosa PA14	> 64
C. freundii DSM30039	64
E. faecalis ATCC29212	16
E. faecalis DSM20478	> 64
E. faecium DSM20477	> 64
S. aureus Newman	> 64
M. luteus DSM1790	64
B. subtilis DSM10	> 64
S. pneumoniae DSM11865	64
**Cell line**	**IC_{50} [µM]**
A-549	> 37
CHO-K1	> 37
HEK-293	> 37
HepG2	> 37

Table S2. Relative shifts of corramycin activity against *M. tuberculosis* strains from different lineages and with different drug resistance profiles. Fold MICs are based on the reference strain *Mtb* H37Rv. Experiments were performed once. BDQ, bedaquiline; CPM, capreomycin; EMB, ethambutol; ETH, ethionamide; INH, isoniazid; LFX, levofloxacin; MOX, moxifloxacin; REMA, Resazurin Microtiter Assay; RIF, rifampicin; STR, streptomycin.

***Mtb* strain/ID**	**Resistance profile**	**Fold MIC**	**MIC Method**
H37Rv	Wild-type	1	REMA/ EUCAST protocol (version 8.2)
Haarlem 2336/02	Clinical isolates of lineage 4 (Euro-American), drug-sensitive	1	REMA
Haarlem 9532/03		2	
Haarlem 4130/02		2	
Beijing 5331/06	Clinical isolates of lineage 2 (East Asian), drug-sensitive	8	
Beijing 1500/03		2	
Beijing 1934/03		2	
Isolate 24002497	Clinical isolates of lineage 4 (Euro-American), MDR, RIF- and INH-resistant	1	EUCAST protocol (version 8.2)
Isolate 22005979		0.5	
Isolate 22005414		1	
Isolate 21002601	Clinical isolate of lineage 2 (East Asian), resistant to INH, EMB, STR and ETH	< 1	
Isolate 21003539	Clinical isolate of lineage 3 (Central Asian Strain (CAS)/Delhi), resistant to MOX and LFX	< 1	
Isolate 21003374	Clinical isolate of lineage 4 (Euro-American), resistant to MOX, LFX, INH, STR and CPM	2	
Isolate 21005057	Clinical isolate of lineage 4, resistant to MOX and LFX	2	
Isolate 23005525	Clinical isolates of lineage 2 (East Asian), MDR, RIF- and INH-resistant	1	
Isolate 21008001		0.5	
Isolate 21007464		1	
H37Rv *rpoB* H445D	RIF mono-resistant	1	REMA
H37Rv *atpE* D28G	BDQ mono-resistant	2	
H37Rv *atpE* E61D		2	
H37Rv *atpE* A63P		2	

Table S3. Mutations in corramycin-resistant *M. tuberculosis* (Erdman) mutants.

Mutant	Locus tag	Gene	Mutation	Description
First-step mutants (*Mtb* Erdman wild-type as parental strain)				
KNC_125	Rv0005	*gyrB*	R482L (cgc/cTc)	DNA gyrase subunit B
	Rv2248	-	G271V (ggc/gTc)	Hypothetical protein
	Rv2512c	-	Q328K (cag/Aag)	Transposase for insertion sequence element IS1081
	Rv2932	*ppsB*	D1122H (gat/Cat)	Phenolpthiocerol synthesis type-I polyketide synthase
KNC_123	Rv0006	*gyrA*	D89E (gac/gaA)	DNA gyrase subunit A
	Rv2512c	-	Q328K (cag/Aag)	Transposase for insertion sequence element IS1081
Second-step mutants (KNC_123 as parental strain)				
KNC_190	Rv0005	*gyrB*	D461E (gac/gaA)	DNA gyrase subunit B
	Rv3511	*PE_PRGS55*	N396D (aac/Gac)	PE-PGRS family protein PE_PGRS55
C20_17[a]	Rv0005	*gyrB*	R482L (cgc/cTc)	DNA gyrase subunit B

[a]Mutant information was determined by Sanger sequencing of *gyrA* and *gyrB*.

Table S4. Activities of corramycin against spontaneous resistant and recombineered *M. tuberculosis* mutants. Fold MICs are based on the respective wild-type strains.

**Genotype**	**Fold MIC**
Mtb Erdman wild-type	1
KNC_125 *gyrB* R482L	6.1
KNC_123 *gyrA* D89E	23.3
KNC_190 *gyrA* D89E, *gyrB* D461E	314.8
C20-17 *gyrA* D89E, *gyrB* R482L	165.9
Mtb pJV53 wild-type	1
Mtb pJV53 *gyrB* D461E	5.9
Mtb pJV53 *gyrB* R482L	3.1
Mtb pJV53 *gyrA* D89E	23.7

KNC strains are spontaneous resistant mutants selected on corramycin.
pJV53 strains are recombineered mutants harboring defined *gyrA* or *gyrB* mutations.

Table S5. Mutations in corramycin-resistant *M. tuberculosis* (H37Ra) mutants selected via serial passaging. Antibiotic susceptibility profiles of the mutants are summarized in Table S6.

Mutant	Locus tag	Gene	Mutation	Description
'Final' mutants harvested at 8.4 µM corramycin pressure				
M2	Rv0006	*gyrA*	L510S (ttg/tCg)	DNA gyrase subunit A
	Rv1819c	*bacA*	162_163insC	Vitamin B_{12} transport ATP-binding protein BacA
	Rv1282c	*oppC*	771delC	Oligopeptide ABC transporter permease OppC
	Rv1339	-	V261M (gtg/Atg)	Hypothetical protein
M3	Rv0005	*gyrB*	R482L (cgc/cTc)	DNA gyrase subunit B
	Rv0878c	-	1307_1308insC	PPE family protein PPE13
	Rv1253	*deaD*	V190F (gtc/Ttc)	ATP-dependent RNA helicase DeaD
M4	Rv1819c	*bacA*	509_510insGC	Vitamin B_{12} transport ATP-binding protein BacA
	Rv1280c	*oppA*	700_701insGC	Oligopeptide ABC transporter substrate-binding lipoprotein OppA
	Rv2396	*aprC*	D190E (gac/gaA)	Acid and phagosome regulated protein AprC
M7	Rv0006	*gyrA*	D89E (gac/gaA)	DNA gyrase subunit A
	Rv1819c	*bacA*	505delA	Vitamin B_{12} transport ATP-binding protein BacA
	Rv1281c	*oppD*	K592Stop (aaa/Taa)	Oligopeptide ABC transporter ATP-binding protein OppD
	Rv2299c	*htpG*	N409T (aac/aCc)	Chaperone protein HtpG
M8	Rv0006	*gyrA*	T80S (acc/Tcc)[a]	DNA gyrase subunit A
M9	Rv0006	*gyrA*	G694D (ggt/gAt)	DNA gyrase subunit A
	Rv1819c	*bacA*	383_384insT	Vitamin B_{12} transport ATP-binding protein BacA
	Rv1283c	*oppB*	241_242insA	Oligopeptide ABC transporter permease OppB
'Progenitor' mutants harvested at 2.5 µM corramycin pressure				
M2p	Rv0006	*gyrA*	L510S (ttg/tCg)	DNA gyrase subunit A
	Rv1339	-	V261M (gtg/Atg)	Hypothetical protein
M3p	Rv0005	*gyrB*	R482L (cgc/cTc)	DNA gyrase subunit B
	Rv0878c	-	1307_1308insC	PPE family protein PPE13
	Rv1253	*deaD*	V190F (gtc/Ttc)	ATP-dependent RNA helicase DeaD
M4p	Rv1819c	*bacA*	509_510insGC	Vitamin B_{12} transport ATP-binding protein BacA
	Rv1280c	*oppA*	700_701insGC	Oligopeptide ABC transporter substrate-binding lipoprotein OppA
M7p	Rv0006	*gyrA*	D89E (gac/gaA)	DNA gyrase subunit A
	Rv2299c	*htpG*	N409T (aac/aCc)	Chaperone protein HtpG

[a]Variant detected in 60% of the reads.

Table S6. Antibiotic susceptibilities of spontaneous *M. tuberculosis* (H37Ra) mutants selected via serial passaging. Note that serial passaging led to loss of the PDIM lipid, resulting in decreased MIC values for corramycin, rifampicin and streptomycin.[5] BDQ, bedaquiline; BLM, bleomycin; CIP, ciprofloxacin; COR, corramycin; EMB, ethambutol; GEP, gepotidacin; INH, isoniazid; LIN, linezolid; MOX, moxifloxacin; RIF, rifampicin; STR, streptomycin.

**Mutant**	**MIC [µg/mL]**										
	**COR**	**CIP**	**MOX**	**GEP**	**RIF**	**EMB**	**INH**	**LIN**	**BDQ**	**STR**	**BLM**
‘Progenitor’ mutants harvested at 2.5 µM corramycin pressure											
WTp	0.0156	1	0.125	8	0.002	4	0.25	1	0.008	0.5	0.0156
M2p	2	2	0.25	8	0.008	4	0.5	1	0.008	1	0.0156
M3p	2	1	0.125	8	0.004	4	0.5	1	0.0156	1	0.0156
M4p	32	1	0.25	8	0.004	2	0.25	2	0.0156	2	–
M7p	16	2	0.25	16	0.004	4	0.5	1	0.0156	1	0.0156
‘Final’ mutants harvested at 8.4 µM corramycin pressure											
WT	0.0156	1	0.125	4	0.002	2	0.5	1	0.008	0.5	0.0156
M2	16	2	0.25	16	0.004	4	0.5	2	0.0156	1	0.125
M3	4	0.25	0.125	4	0.004	2	0.5	1	0.008	1	–
M4	32	1	0.125	4	0.002	2	0.5	1	0.008	1	1
M7	> 64	1	0.25	8	0.004	2	0.5	1	0.008	2	1
M8	4	2	0.25	16	0.004	4	0.5	2	0.008	2	–
M9	32	1	0.125	8	0.004	2	0.25	1	0.0156	4	1

Table S7. Data collection and refinement statistics.

***Mtb*Gyr-Corramycin**		
PDB code	9QUT	
Main EMDB code	EMD-53381	
**Data collection and processing**		
Microscope	ThermoFisher Krios3 G4	
Detector	Falcon 4i	
Magnification	165 000x	
Voltage (kV)	300	
Total Dose (e^-/Å^2)	50	
Pixel Size (Å)	0.731	
Defocus Range (µm)	-0.7 to -2.0	
Micrographs (no.) (0°, 30°)	9,427	9,822
Total particles images (no.) (0°, 30°)	2,428,348	2,545,205
Final Particle Number	97,446	
**Refinement**		
Resolution (Å)<br>FSC 0.143 (unmasked/masked)	3.60 Å/2.80 Å	
Map sharpening *B* factor (Å^2)	No sharpening	
Model composition<br>Non-hydrogen atoms<br>Protein residues<br>Nucleotides<br>Ligands	 12372<br>1446<br>48<br>2	
Mean B factors (Å^2)<br>Protein<br>Nucleotide<br>Ligands	 80<br>97<br>86	
R.m.s. deviations<br>Bond lengths (Å)<br>Bond angles (°)	 0.004<br>0.607	
Validation<br>MolProbity score<br>All-atom clashscore	 2.3<br>11	
Ramachandran plot<br>Favoured (%)<br>Allowed (%)<br>Disallowed (%)	 94.7<br>5.14<br>0.14	

Table S8. Hydrogen bond interactions between corramycin and residues from GyrA and GyrB subunits.

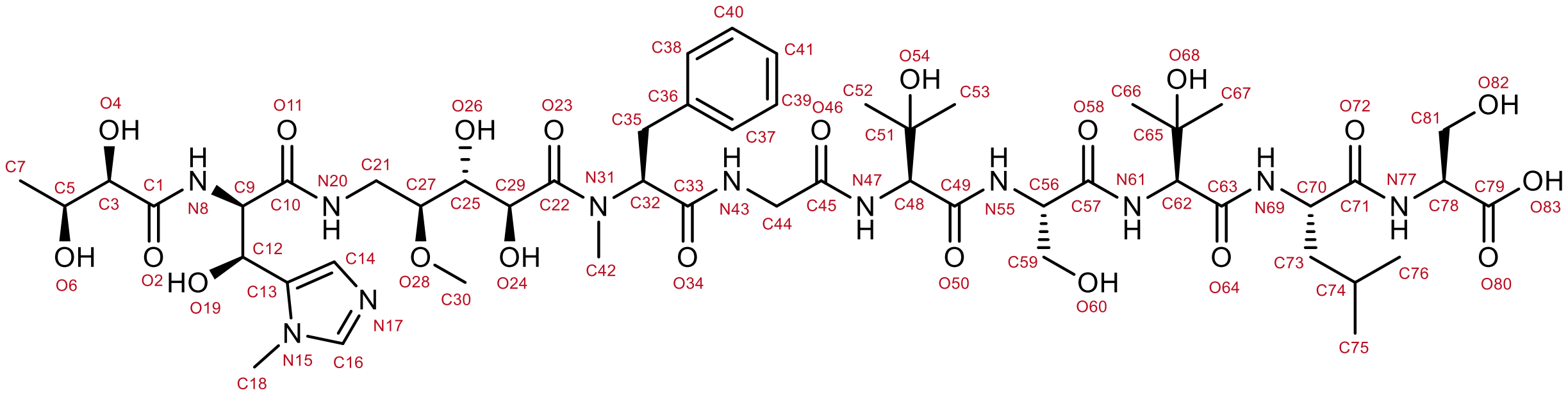

Residue	Position (chain)	Atom	COR atom (chain I)	Distance (Å)
Met	81 (C)	SD	N61	2.9
Asp	89 (C)	N	O50	3.2
Asp	89 (C)	OD1	N47	2.9
Asp	89 (C)	OD1	O54	3.1
Glu	459 (D)	OE2	N17	2.8
Asp	461 (D)	N	O24	3.5
Asp	532 (D)	OD2	N17	2.8
Asp	534 (D)	OD1	N17	3.4
Lys	611 (D)	NZ	O58	3.8
Leu	613 (D)	N	O6	3.4
Gly	614 D)	N	O4	3.0
			**COR atom (chain J)**	
Met	81 (A)	SD	N61	3.0
Asp	89 (A)	N	O50	3.3
Asp	89 (A)	OD1	N47	3.0
Asp	89 (A)	OD1	O54	2.9
Glu	459 (B)	OE2	N17	2.8
Asp	461 (B)	N	O24	3.5
Asp	532 (B)	OD2	N17	2.8
Asp	534 (B)	OD1	N17	3.4
Leu	613 (B)	N	O6	3.5
Gly	614 (B)	N	O4	3.0

Table S9. Oligonucleotides used in this study.

Oligo	Method	Sequence (5'-3')
gyrB D461E-F	Site-directed recombineering in *Mtb* Erdman[a]	CGCGCAAGTCCGAACTGTATGTCGTAGAAGGTGA**A**TCGG CCGGCGGTTCTGCAAAAAGCGGTCGCGATTC
gyrB D461E-R		GAATCGCGACCGCTTTTTGCAGAACCGCCGGCCGA**T**TCAC CTTCTACGACATACAGTTCGGACTTGCGCG
gyrB R482L-F		CGCGATTCGATGTTCCAGGCGATACTTCCGCTGC**T**CGGCA AGATCATCAATGTGGAGAAAGCGCGCATCG
gyrB R482L-R		CGATGCGCGCTTTCTCCACATTGATGATCTTGCCG**A**GCAG CGGAAGTATCGCCTGGAACATCGAATCGCG
gyrA D89E-F		CCGAGACCATGGGCAACTACCACCCGCACGGCGA**A**GCGT CGATCTACGACAGCCTGGTGCGCATGGCCCA
gyrA D89E-R		TGGGCCATGCGCACCAGGCTGTCGTAGATCGACGC**T**TCG CCGTGCGGGTGGTAGTTGCCCATGGTCTCGG
ME-73b	Cryo-EM	TTCGGCGAGAAGCAGGCCATTATCGCCGGCATGGCGGCC GACGCGCTGGGCTACGTCTTGCTGGCGTTCGCGA
ME-77b		CGCGAGGCTGGATGGCCTTCCCCATTATGATTCTTCTCGC TTCCGGCGGCATCGGGATGCCCGCGTTGCAGGCCATG

[a]Bold letters indicate amino acid substitution compared to wild-type.

Table S10. Chemical shifts of corramycin in methanol-D3 at 300 K. Chemical shifts were referenced to the solvent signals (¹H: 3.30 ppm, ¹³C: 49.00 ppm).

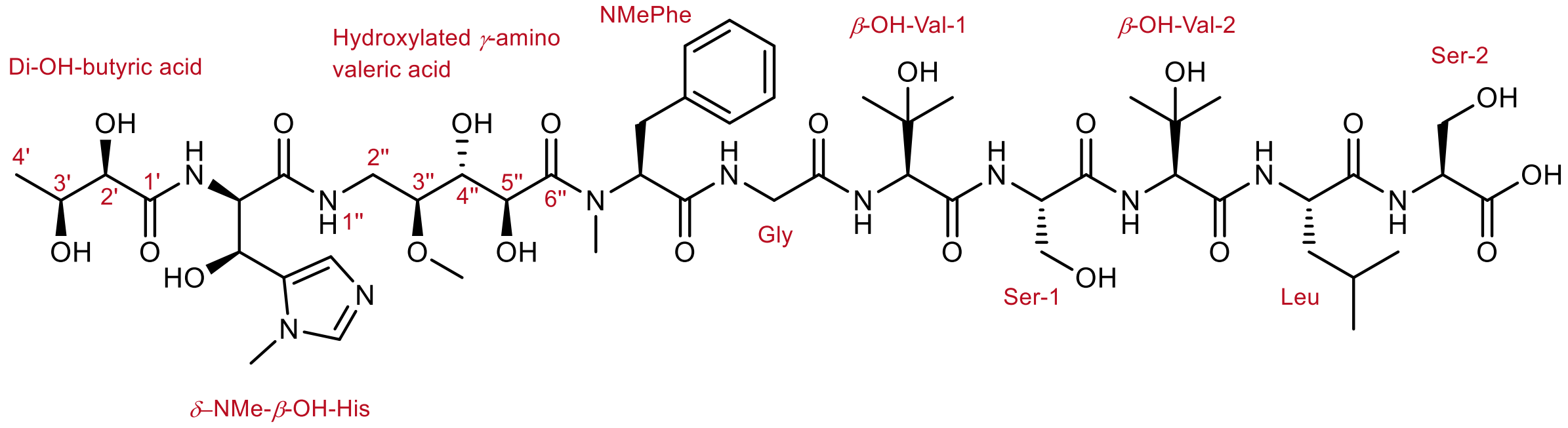

	δ (¹H) [ppm]	δ (¹³C) [ppm]
**Di-OH-butyric acid**		
1’	-	177.19
2’	3.80	76.18
3’	3.84	69.29
4’	1.10	19.78
***δ*-NMe-*β*-OH-His**		
NH	8.02	-
α	5.26	56.45
β	5.01	67.19
γ	-	134.36
δ	7.39	121.14
δ-NMe	4.02	35.30
ε	8.68	138.03
C’	-	171.72
**Hydroxylated *γ*-amino valeric acid**		
1”	8.35	-
2”	3.91/3.32	38.76
3”	3.54	79.88
3”-OMe	3.23	57.25
4”	4.13	71.01
5”	4.39	71.01
6”	-	174.70
**NMePhe**		
NMe	2.95	32.14
α	5.46	60.47
β	3.50/2.96	34.27
γ	-	138.87
δ	7.23	130.04
ε	7.23	129.47
ζ	7.16	127.60
C’	-	172.10
**Gly**		
NH	7.77	-
α	4.37/3.75	43.47
C’	-	171.11
***β*-OH-Val-1**		
NH	8.31	-
α	4.81	61.19
β	-	73.34

γ	1.21	25.51
γ	1.19	28.19
C’	-	172.14
**Ser-1**		
NH	8.46	-
α	4.53	57.19
β	3.86/3.79	62.93
C’	-	172.33
**β-hydroxy-Val-2**		
NH	8.05	-
α	4.41	62.12
β	-	72.92
γ	1.21	26.37
γ	1.25	27.88
C’	-	172.21
**Leu**		
NH	8.17	-
α	4.48	53.22
β	1.64	41.54
γ	1.70	25.79
δ	0.93	23.55
δ	0.90	21.79
C’	-	174.70
**Ser-2**		
NH	8.14	-
α	4.44	56.40
β	3.88/3.81	62.90
C’	-	173.26

Table S11. Distance constraints used for molecular dynamics calculations and energy minimizations. Exp. Dist., experimental distance; Lower, lower limit of the distance range used for calculations (Exp. Dist. minus 10%); Upper, upper limit of the distance range used for calculations (Exp. Dist. plus 10% and pseudoatom correction); Av. Dist., average distance over the 10 energy minimized structures; Av. Viol., average violation of the experimental constraints over the 10 energy minimized structures.

Atom 1	Atom 2	Exp. Dist.	Lower	Upper	Av. Dist.	Av. Viol.
VAL3.HA	HIS.HA	2.97	2.67	3.27	2.64	0.03
HIS.HA	VAL3.QG2	4.27	3.85	5.70	4.55	0.00
HIS.HA	VAL3.QG1	4.19	3.77	5.60	3.43	0.34
VAL3.HA	HIS.DQNME	3.12	2.81	4.43	3.10	0.00
SER4.H	HIS.DQNME	3.63	3.27	4.99	2.65	0.62
1".NH	HIS.HA	2.40	2.16	2.64	2.23	0.00
VAL3.HA	1".NH	4.10	3.69	4.51	3.88	0.00
1".NH	HIS.DQNME	4.12	3.71	5.54	3.63	0.08
4".H	1".NH	2.90	2.61	3.19	2.45	0.16
HIS.H	HIS.DQNME	4.12	3.70	5.53	5.62	0.09
HIS.H	2'.H	3.08	2.77	3.39	3.60	0.21
5".H	PHE.QNME	2.19	1.97	3.40	3.09	0.00
GLY2.H	3".QOME	3.00	2.7	4.29	3.09	0.00
GLY2.H	3".H	4.51	4.06	4.96	4.59	0.00
PHE1.HA	3".QOME	3.90	3.51	5.29	4.52	0.00
PHE1.HA	GLY2.H	3.19	2.87	3.51	2.75	0.12
GLY2.H	HIS.DQNME	4.52	4.07	5.98	4.69	0.00
HIS.HA	HIS.DQNME	2.45	2.21	3.70	3.72	0.02
HIS.HA	3".QOME	4.54	4.08	5.99	4.66	0.00
VAL3.H	PHE.QNME	4.37	3.93	5.81	5.53	0.00
1".NH	PHE.QNME	4.11	3.70	5.52	4.08	0.00
GLY2.H	PHE.QNME	2.82	2.54	4.10	3.11	0.00
4".H	PHE.QNME	2.32	2.09	3.55	2.76	0.00
SER4.HB1	HIS.EH	4.20/4.22	3.78	4.64	4.28	0.00
SER4.HA	HIS.DQNME	4.34	3.91	5.78	4.64	0.00
VAL3.HA	SER4.H	2.63	2.37	2.89	2.77	0.00
SER4.H	HIS.HA	4.48	4.03	4.93	4.96	0.03
5".H	1".NH	4.54	4.09	5.00	4.86	0.00
1".NH	3".QOME	3.51	3.16	4.86	3.19	0.00
PHE.QNME	HIS.DQNME	3.10	2.79	5.41	3.24	0.00
GLY2.HA2	VAL3.H	2.69	2.42	2.96	2.39	0.03
GLY2.HA1	VAL3.H	2.63	2.37	2.89	3.04	0.15
VAL3.H	HIS.DQNME	4.93	4.44	6.43	4.80	0.00
HIS.HA	PHE.QNME	3.91	3.52	5.30	5.53	0.23
VAL3.H	3".QOME	5.23	4.71	6.76	6.63	0.00
SER4.HB2	HIS.EH	4.20/4.22	3.78	4.64	5.08	0.44
VAL5.HA	LEU6.H	2.38	2.14	2.62	2.52	0.00
LEU6.H	VAL5.QG2	3.72	3.35	5.10	4.98	0.00
LEU6.H	VAL5.QG1	3.81	3.43	5.19	5.25	0.06
LEU6.H	LEU6.QD1	4.85	4.37	6.34	5.11	0.00
LEU6.H	LEU6.QD2	4.97	4.47	6.47	5.30	0.00
LEU6.HA	SER7.H	2.57	2.31	2.83	2.28	0.03
LEU6.HB1	SER7.H	3.69/3.89	3.32	4.28	4.51	0.23
LEU6.HB2	SER7.H	3.69/3.89	3.32	4.28	4.47	0.19
SER4.HA	VAL5.H	2.53	2.28	2.78	2.37	0.00
HIS.H	4'.QME	4.17	3.74	5.59	4.70	0.00
HIS.H	3'.H	3.33	3.00	3.66	3.73	0.07
HIS.HB	HIS.H	2.87	2.59	3.16	2.94	0.00
HIS.HA	HIS.H	2.96	2.66	3.25	2.88	0.00
HIS.DH	HIS.H	3.99	3.59	4.39	4.28	0.00
HIS.HA	4'.QME	4.98	4.48	6.47	6.48	0.01
HIS.HB	HIS.DH	2.84	2.56	3.13	3.81	0.68
SER4.H	SER4.HB2	2.97/3.02	2.67	3.32	2.49	0.18

SER4.H	SER4.HB1	2.97/3.02	2.67	3.32	2.97	0.00
1".NH	VAL3.QG1	3.64	3.29	5.01	5.47	0.47
1".NH	VAL3.QG2	4.16	3.75	5.58	5.63	0.05
VAL3.H	VAL3.QG2	2.91	2.62	4.20	3.23	0.00
VAL3.H	VAL3.QG1	3.38	3.04	4.72	4.69	0.00
SER4.H	VAL3.QG2	3.68	3.31	5.04	5.25	0.21
SER4.H	VAL3.QG1	3.91	3.52	5.30	5.24	0.00
GLY2.H	VAL3.QG2	4.86	4.37	6.34	5.20	0.00
GLY2.H	VAL3.QG1	5.16	4.64	6.68	6.75	0.07
VAL3.HA	VAL3.QG2	2.76	2.48	4.04	3.36	0.00
VAL3.HA	VAL3.QG1	2.55	2.29	3.80	2.74	0.00
SER4.HA	VAL3.QG1	4.73	4.26	6.21	6.29	0.08
LEU6.HA	LEU6.QD1	2.66	2.39	3.93	2.95	0.00
LEU6.HA	LEU6.QD2	3.33	3.00	4.66	4.65	0.00
3".H	1".NH	3.48	3.13	3.83	3.62	0.00
SER4.HB2	VAL5.H	3.41/3.54[a]	3.07	3.89	4.00	0.11
SER4.HB1	VAL5.H	3.41/3.54	3.07	3.89	3.54	0.00
HIS.HB	VAL3.QG1	4.54	4.09	5.99	4.07	0.02
HIS.HB	VAL3.QG2	4.62	4.16	6.09	5.95	0.00
HIS.HB	PHE.QNME	4.22	3.80	5.64	5.62	0.00
HIS.HB	HIS.DQNME	2.64	2.38	3.91	2.77	0.00
4".H	HIS.HB	4.17	3.75	4.59	4.80	0.22
HIS.HB	3".QOME	4.57	4.11	6.03	6.57	0.55
GLY2.H	1".NH	3.89	3.50	4.28	3.60	0.00
1".NH	HIS.HB	2.95	2.65	3.24	3.57	0.33
GLY2.H	HIS.HA	4.99	4.49	5.49	4.90	0.00
VAL3.H	SER4.H	3.25	2.93	3.58	2.75	0.18
SER4.H	SER4.HA	2.99	2.69	3.29	2.75	0.00
SER7.H	VAL5.QG2	4.57	4.11	6.02	5.83	0.00
SER7.H	VAL5.QG1	4.03	3.63	5.43	5.48	0.05
VAL5.H	VAL5.QG2	3.36	3.03	4.70	4.70	0.00
VAL5.H	VAL5.QG1	2.88	2.59	4.16	4.00	0.00
SER4.HA	VAL5.QG2	4.61	4.15	6.07	6.74	0.67
VAL5.HA	VAL5.QG2	2.57	2.31	3.83	2.95	0.00
VAL5.HA	VAL5.QG1	2.71	2.44	3.98	3.90	0.00
5".H	3".QOME	4.21	3.79	5.64	5.75	0.11
4".H	3".QOME	3.21	2.88	4.53	4.29	0.00
GLY2.HA1	3".QOME	3.10	2.79	4.41	4.47	0.06
GLY2.HA2	PHE.QNME	3.47	3.12	4.82	4.54	0.00
GLY2.HA1	PHE.QNME	4.42	3.98	5.86	5.15	0.00
GLY2.HA1	VAL3.QG2	3.98	3.58	5.38	3.56	0.02
GLY2.HA1	VAL3.QG1	4.12	3.70	5.53	6.04	0.51
VAL3.H	SER4.HA	4.65	4.19	5.12	4.47	0.00
GLY2.H	VAL3.H	3.99	3.59	4.38	4.45	0.07
LEU6.H	LEU6.HA	2.95	2.65	3.24	2.68	0.00
SER7.H	SER7.HB1	3.26/3.38	2.93	3.72	2.91	0.02
SER7.H	SER7.HB2	3.26/3.38	2.93	3.72	3.68	0.00
VAL5.H	VAL5.HA	2.94	2.64	3.23	2.36	0.28
GLY2.HA1	GLY2.H	2.61	2.35	2.87	2.55	0.00
GLY2.HA2	GLY2.H	2.88	2.59	3.16	3.01	0.00
GLY2.H	4".H	3.38	3.04	3.72	3.91	0.19
PHE1.HA	4".H	5.35	4.81	5.88	4.98	0.00
PHE1.HA	HIS.DQNME	4.87	4.38	6.36	6.64	0.28
4".H	HIS.HA	4.04	3.64	4.44	4.43	0.00

[a]In cases where ROEs to both protons of a methylene group are observed (and no diastereotopic assignment has been achieved), the lower limit of the shorter distance and the upper limit of the longer distance has been used for both constrains.

References

1. Blower, T.R., Williamson, B.H., Kerns, R.J., and Berger, J.M. (2016). Crystal structure and stability of gyrase–fluoroquinolone cleaved complexes from *Mycobacterium tuberculosis*. Proc. Natl. Acad. Sci. U.S.A. *113*, 1706–1713. https://doi.org/10.1073/pnas.1525047113.

2. Adam, S., Fries, F., von Tesmar, A., Rasheed, S., Deckarm, S., Sousa, C.F., Reberšek, R., Risch, T., Mancini, S., Herrmann, J., et al. (2024). The Peptide Antibiotic Corramycin Adopts a β-Hairpin-like Structure and Is Inactivated by the Kinase ComG. J. Am. Chem. Soc. *146*, 8981–8990. https://doi.org/10.1021/jacs.3c13208.

3. Michalczyk, E., Hommernick, K., Behroz, I., Kulike, M., Pakosz-Stępień, Z., Mazurek, L., Seidel, M., Kunert, M., Santos, K., von Moeller, H., et al. (2023). Molecular mechanism of topoisomerase poisoning by the peptide antibiotic albicidin. Nat Catal *6*, 52–67. https://doi.org/10.1038/s41929-022-00904-1.

4. Gedeon, A., Yab, E., Dinut, A., Sadowski, E., Capton, E., Dreneau, A., Petit, J., Gioia, B., Piveteau, C., Djaout, K., et al. (2024). Molecular mechanism of a triazole-containing inhibitor of Mycobacterium tuberculosis DNA gyrase. iScience *27*, 110967. https://doi.org/10.1016/j.isci.2024.110967.

5. Mulholland, C.V., Wiggins, T.J., Cui, J., Vilchèze, C., Rajagopalan, S., Shultis, M.W., Reyes-Fernández, E.Z., Jacobs, W.R., and Berney, M. (2024). Propionate prevents loss of the PDIM virulence lipid in Mycobacterium tuberculosis. Nat Microbiol *9*, 1607–1618. https://doi.org/10.1038/s41564-024-01697-8.
